# Zero-shot instance segmentation for plant phenotyping in vertical farming with foundation models and VC-NMS

**DOI:** 10.3389/fpls.2025.1536226

**Published:** 2025-05-05

**Authors:** Qin-Zhou Bao, Yi-Xin Yang, Qing Li, Hai-Chao Yang

**Affiliations:** ^1^ College of Mathematics and Computer Science, Dali University, Dali, China; ^2^ School of Electronic Information, Xijing University, Xian, China

**Keywords:** segment anything, zero-shot, instance segmentation, prompt augmentation, foundation models

## Abstract

**Introduction:**

Image instance segmentation is essential for plant phenotyping in vertical farms, yet the diversity of plant types and limited annotated image data constrain the performance of traditional supervised techniques. These challenges necessitate a zero-shot approach to enable segmentation without relying on specific training data for each plant type.

**Methods:**

We present a zero-shot instance segmentation framework combining Grounding DINO and the Segment Anything Model (SAM). To enhance box prompts, Vegetation Cover Aware Non-Maximum Suppression (VC-NMS) incorporating the Normalized Cover Green Index (NCGI) is used to refine object localization by leveraging vegetation spectral features. For point prompts, similarity maps with a max distance criterion are integrated to improve spatial coherence in sparse annotations, addressing the ambiguity of generic point prompts in agricultural contexts.

**Results:**

Experimental validation on two test datasets shows that our enhanced box and point prompts outperform SAM’s everything mode and Grounded SAM in zero-shot segmentation tasks. Compared to the supervised method YOLOv11, our framework demonstrates superior zero-shot generalization, achieving the best segmentation performance on both datasets without target-specific annotations.

**Discussion:**

This study addresses the critical issue of scarce annotated data in vertical farming by developing a zero-shot segmentation framework. The integration of domain-specific indices (NCGI) and prompt optimization techniques provides an effective solution for plant phenotyping, highlighting the potential of weakly supervised models in agricultural computer vision where extensive manual annotation is impractical.

## 1 Introduction

Vertical farming, a type of indoor agriculture, has gained significant attention in recent years due to its potential for efficient and sustainable plant production (Benke and Tomkins, 2017). In vertical farms, plants are grown in stacked layers or vertically inclined structures, utilizing controlled environments and artificial lighting systems (Al-Kodmany, 2018). Each agricultural unit functions as an independent ecosystem that creates an optimal environment for plant growth. By manipulating the spectrum of light, temperature, humidity, pH, and nutrient levels, researchers can ensure that each plant exhibits optimal taste, color, and nutritional quality (Franchetti et al., 2019). Accurate measurement of plant traits is essential for optimized resource utilization and maximized yield in closed and controlled environments. Plant phenotyping is critical for accurately measuring plant traits. The majority of currently used phenotyping techniques are destructive and time-consuming. Recently, the development of various sensors and imaging platforms for rapid and efficient quantitative measurement of plant traits has become the mainstream approach in plant phenotyping studies (Abebe et al., 2023).

The first step in extracting image-based plant growth phenotypes involves applying an image segmentation algorithm to separate plant leaves from the background in RGB images (Guo et al., 2023). Geometric features, including leaf projection area, volume, and plant height, can then be derived. Among segmentation techniques, the thresholding method is considered the simplest approach. Its core principle is to classify image pixels based on their grayscale values and one or more predefined thresholds. This approach works well for plant images against simple backgrounds. Li et al. (2020) proposed a non-invasive approach to measure plant growth characteristics using smartphone-based image analysis. By employing the Otsu thresholding method (Otsu, 1979) to extract plant leaf area, the researchers achieved non-destructive monitoring of relative growth rates. Chang et al. (2021) converted RGB images to the HSV color space and defined threshold ranges to distinguish target objects from the background. They further applied noise filtering and edge detection to delineate lettuce outlines and extract leaf areas for growth rate evaluation. Although the thresholding method is simple and fast, it requires predefined or adaptively determined optimal thresholds. Consequently, its performance degrades in complex scenes or under varying lighting conditions. In contrast to thresholding, traditional machine learning (ML) techniques like Random Forest, Support Vector Machines (SVMs), and clustering algorithms better handle complex scenes. Lee et al. (2018) applied the Random Forest to extract growth phenotypic traits such as plant height, width, area, and leaf area. Zhou et al. (2017) used a k-means-based color clustering algorithm to segment plant images and extract growth phenotypes such as projected leaf area, leaf circumference, compactness, leaf count, and daily relative growth rate. Guo et al. (2021) developed KAT4IA, a self-supervised pipeline. This method uses k-means clustering on greenhouse images to generate training data, enabling maize plant height extraction in image-based phenotyping systems. Despite their adaptability, traditional ML methods often require manual feature extraction and preprocessing, which limit model scalability and significantly increase both the complexity and time required for analysis (Zhang et al., 2020).

Deep learning (DL) approaches automatically learn feature representations through neural networks, eliminating manual feature design and enabling complex plant phenotype extraction. In recent years, DL techniques have been increasingly utilized for plant growth phenotype analysis. Reyes-Yanes et al. (2020) employed the MASK R-CNN image segmentation method to extract features such as side-view area, height, width, side-view centroid, and top-view area of lettuce, facilitating real-time monitoring of plant growth rates. Trivedi and Gupta (2021) introduced a U-Net architecture based on deep learning for the automatic segmentation of plant images, calculating the leaf area percentage as a growth index to monitor plant growth. This approach efficiently processes plant images, delivers precise leaf segmentation results, and evaluates plant growth status through leaf area percentage calculations. Similarly, Lin et al. (2022) and Cardenas-Gallegos et al. (2024) implemented U-Net to achieve automatic segmentation of plant leaves and background. Khan and Jensen (2025) trained YOLOv11 on a combined multi-crop dataset to extract crop phenotypes, demonstrating that a unified model trained on a combined multi-crop dataset can outperform crop-specific models.

In indoor vertical farms utilizing hydroponics, plant images with relatively simple backgrounds can be effectively processed using the thresholding method to separate plants from the background (Zhang et al., 2020). However, for plant images involving growth media such as clay particles, perlite, or rock wool (Wong et al., 2020), the background becomes more complex. In such cases, the thresholding method is insufficient to accurately separate plants from the background, making deep learning methods a more suitable and effective alternative (Reyes-Yanes et al., 2020). Vertical farms optimize space utilization through vertically stacked planting systems, enabling more plants to grow in limited areas while supporting diverse plant varieties. However, during image segmentation, high plant densities, complex lighting conditions, and the simultaneous cultivation of diverse plant types in vertical farms pose significant challenges to deep learning methods (Hwang et al., 2022). Consequently, vertical farms require models with robust generalization capabilities that can adapt to various plant varieties, lighting conditions, and planting methods in a zero-shot manner.

To address training data scarcity, future research directions should encompass data augmentation techniques, self-supervised learning methods, and the generation of synthetic training data (Liu and Xu, 2023). The Segmentation Anything Model (SAM) (Kirillov et al., 2023) is a powerful and flexible large image segmentation model trained on a dataset containing 11 million images and over 1 billion masks, enabling general segmentation capabilities. SAM can automatically segment new images and leverages point, box, and mask prompts to enhance segmentation performance, making it a viable solution for zero-shot image segmentation tasks in vertical farming. However, recent studies have shown that SAM exhibits limited performance in domain-specific tasks (Chen et al., 2023), such as medical image segmentation (Ma and Wang, 2023; Wu et al., 2023), where segmentation performance largely depends on the quantity and quality of prompts (Deng et al., 2023a). SAM’s training dataset primarily comprises natural images, which typically exhibit clear edge information and strong distinctions. In contrast, agricultural images are often captured in complex field environments with low contrast between targets and backgrounds, diverse agricultural backgrounds, and uneven lighting conditions with shadows. As a result, SAM underperforms in agricultural image segmentation tasks involving such challenging scenarios (Li et al., 2023). To validate this limitation, we tested SAM on plant images with complex backgrounds and uneven lighting in vertical farms. The results confirmed that SAM cannot perfectly “segment anything”. Improving the performance of large models for downstream tasks often involves fine-tuning SAM by retraining the parameters of its Mask-Decoder using new datasets (Zhang et al., 2023d). However, this approach is unsuitable for zero-shot image segmentation required in vertical farming scenarios. Adapters offer an efficient method for fine-tuning large models. By introducing a small number of trainable parameters, adapters allow models to quickly adapt to new tasks while keeping most parameters unchanged (Li et al., 2023). To address SAM’s limitations in agricultural image segmentation, the Agricultural SAM Adapter (ASA) was proposed. ASA incorporates agricultural expertise into the segmentation model by training a small subset of parameters, significantly improving segmentation performance in complex agricultural scenes and offering a novel approach for zero-shot image segmentation in agriculture. Nevertheless, ASA still requires a small amount of training data and does not fully satisfy the need for zero-shot segmentation across diverse plant species in vertical farms. Prompts play a crucial role in SAM’s segmentation performance, acting as an attention mechanism (Zhang et al., 2023c). Enhancing prompts provides additional information to improve SAM’s accuracy. Deng et al. (2023b) proposed a Multi-box prompt augmentation strategy to enhance SAM’s performance in medical image segmentation. Similarly, Dai et al. (2023) compared random sampling, the max entropy criterion, and the max distance criterion, concluding that their proposed Saliency point prompt method enhanced SAM’s segmentation performance across multiple datasets. Ren et al. (2024) integrated GroundingDINO with SAM, naming it “Grounded SAM”, and explored its potential across different tasks. However, Ren et al. only applied box_threshold to filter redundant bounding boxes generated by GroundingDINO without utilizing point prompts, leaving room for further improvement. To systematically evaluate these approaches, we reviewed the relevant literature in recent years and tabulated the results (**Table 1**).

**Table 1:** Recent advances in agricultural image segmentation techniques.

Core Method	Publication Year	Reference
Machine Learning		
random forest	2017	(Lee et al., 2018)
k-means	2018	(Zhou et al., 2017)
Otsu	2020	(Li et al., 2020)
HSV	2021	(Chang et al., 2021)
KAT4IA (k-means)	2021	(Guo et al., 2021)
Deep Learning		
MASK R-CNN	2020	(Reyes-Yanes et al., 2020)
U-Net	2021,2022,2024	(Trivedi and Gupta, 2021) (Lin et al., 2022) (Cardenas-Gallegos et al., 2024)
SAM (fine-tuning)	2023	(Zhang et al., 2023d)
ASA(SAM Adapter)	2023	(Li et al., 2023)
SAM (Multi-box)	2023	(Deng et al., 2023b)
SAM (Saliency)	2023	(Dai et al., 2023)
Grounded SAM	2024	(Ren et al., 2024)
YOLOv11	2025	(Khan and Jensen, 2025)

Compared with traditional agriculture, vertical farming provides more constrained space between plants while accommodating a wider variety of plant species, which poses significant challenges for image segmentation. Different plant types display substantial variations in shape, size, and other traits, making traditional image segmentation methods insufficient. Moreover, in commercial agriculture, high planting density leads to occlusion between adjacent plants. This issue is particularly pronounced in leafy green production, where canopies often merge into nearly continuous structures, significantly increasing the complexity of segmentation tasks (Buxbaum et al., 2022). Additional factors, including uneven lighting and complex backgrounds, further exacerbate the difficulties of image segmentation in vertical farms. While SAM has demonstrated strong zero-shot segmentation capabilities across various plant varieties, its performance degrades when processing images with uneven lighting, diverse planting methods, and complex backgrounds commonly found in vertical farms. To address these challenges, this study leverages SAM as the core image segmentation model and proposes a prompt generation method tailored for vertical farm images. The objective is to effectively overcome the challenges in vertical farm image segmentation and achieve zero-shot instance segmentation for multiple plant varieties using SAM.

This study makes the following key contributions:

1. A zero-shot plant image instance segmentation framework integrating Grounding DINO and Segment Anything Model (SAM) is proposed. This framework tackles visual challenges such as plant diversity, complex lighting, and dense planting. By using text prompts as input, it achieves fully automated plant instance segmentation without requiring image annotations.
2. Vegetation Cover-Aware Non-Maximum Suppression (VC-NMS) algorithm based on the Normalized Cover Green Index (NCGI) is introduced. This algorithm effectively addresses the limitations of traditional NMS in plant image processing and significantly enhances the Average Precision of processed bounding boxes.
3. Our framework employs Grounding DINO combined with VC-NMS to generate bounding box prompts. These prompts are further refined by generating precise positive and negative point prompts within the bounding boxes using the similarity maps and max distance criterion, while determining the optimal number of positive and negative point prompts. Finally, both the refined box prompts and point prompts are input into SAM to enable automated instance segmentation.

## 2 Materials and methods

### 2.1 Data acquisition and DateSet

**Figure 1:**
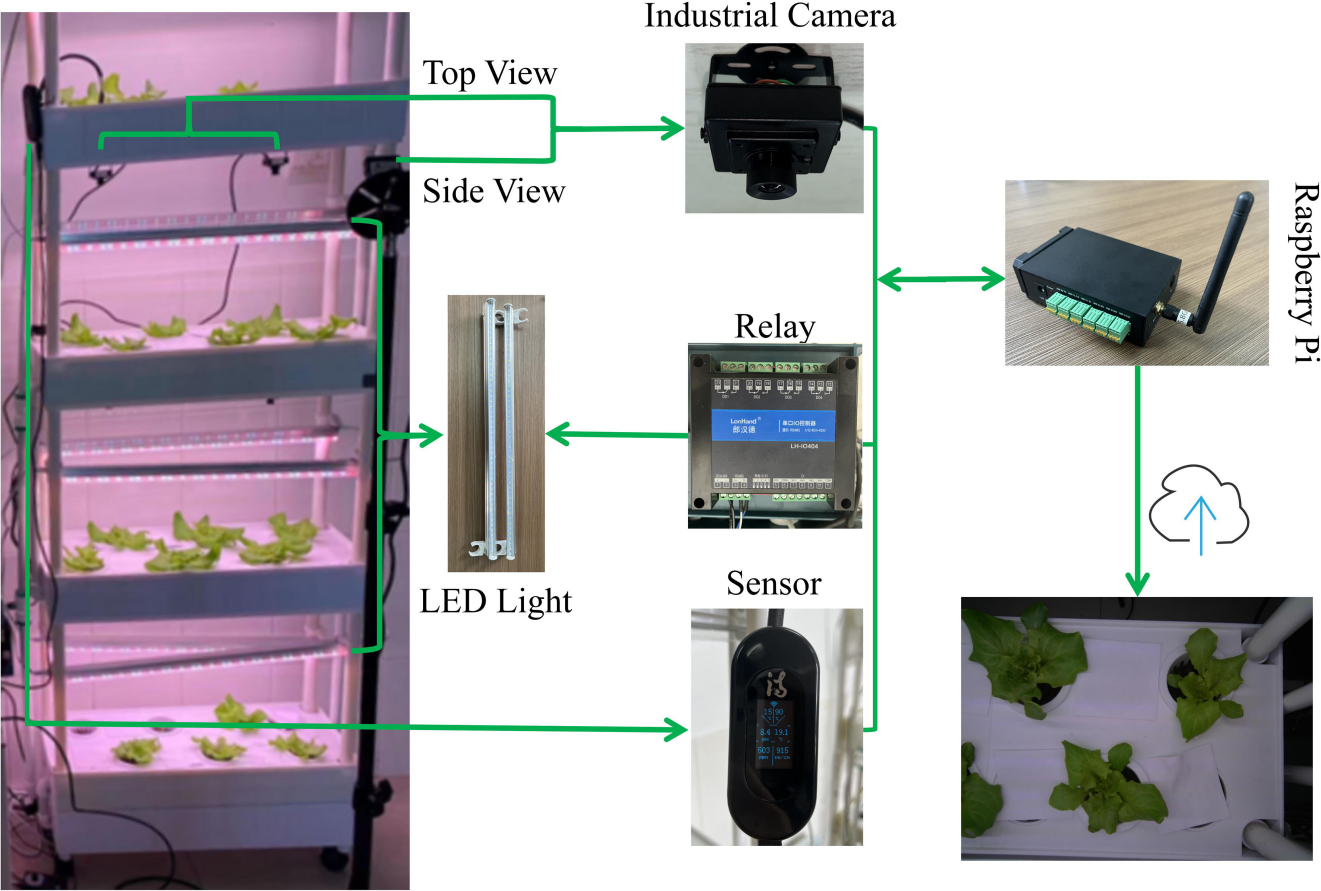
Intelligent vertical farm system. The Raspberry Pi functions as the central control unit, scheduling the switching of LED plant tissue culture lights via relays. It also manages industrial cameras for periodic image capture while synchronously recording sensor data, with all data automatically uploaded to the cloud.

We utilized a multi-layer hydroponic planting machine consisting of four layers, each with 12 holes designed to hold planting baskets. A water circulation system was integrated to ensure consistent water flow from the tank across all layers. Based on this planting system, we developed an intelligent vertical farming system (**Figure 1**) comprising hardware components such as a Raspberry Pi, relays, plant LED lights, sensors, and industrial cameras. A Raspberry Pi-based control and data acquisition software was implemented to enable automated monitoring and control of the vertical farm. Each layer was equipped with either two 5 megapixel (MP) 90° diagonal field of view (FOV) industrial RGB cameras or one 12 MP 130° diagonal FOV industrial RGB camera. Notably, the imaging system operated with auto-white-balance under controlled mixed illumination conditions, including programmable LED photoperiods (16h light/8h dark cycles) and regulated daylight exposure through laboratory windows. This illumination variability triggered continuous white-balance recalibrations, causing observable shifts between red and purple color effects despite stable LED illumination. Sensors collected environmental parameters, including air temperature, air humidity, water temperature, pH, total dissolved solids (TDS), and electrical conductivity (EC). The Raspberry Pi controlled the industrial cameras for scheduled image capture, taking one image every 30 minutes and automatically uploading it to the cloud. Each camera captured approximately 20 images daily. Simultaneously, the Raspberry Pi gathered sensor data at the moment of image capture and uploaded it to the cloud, ensuring precise alignment between images and corresponding environmental data.

**Figure 2:**
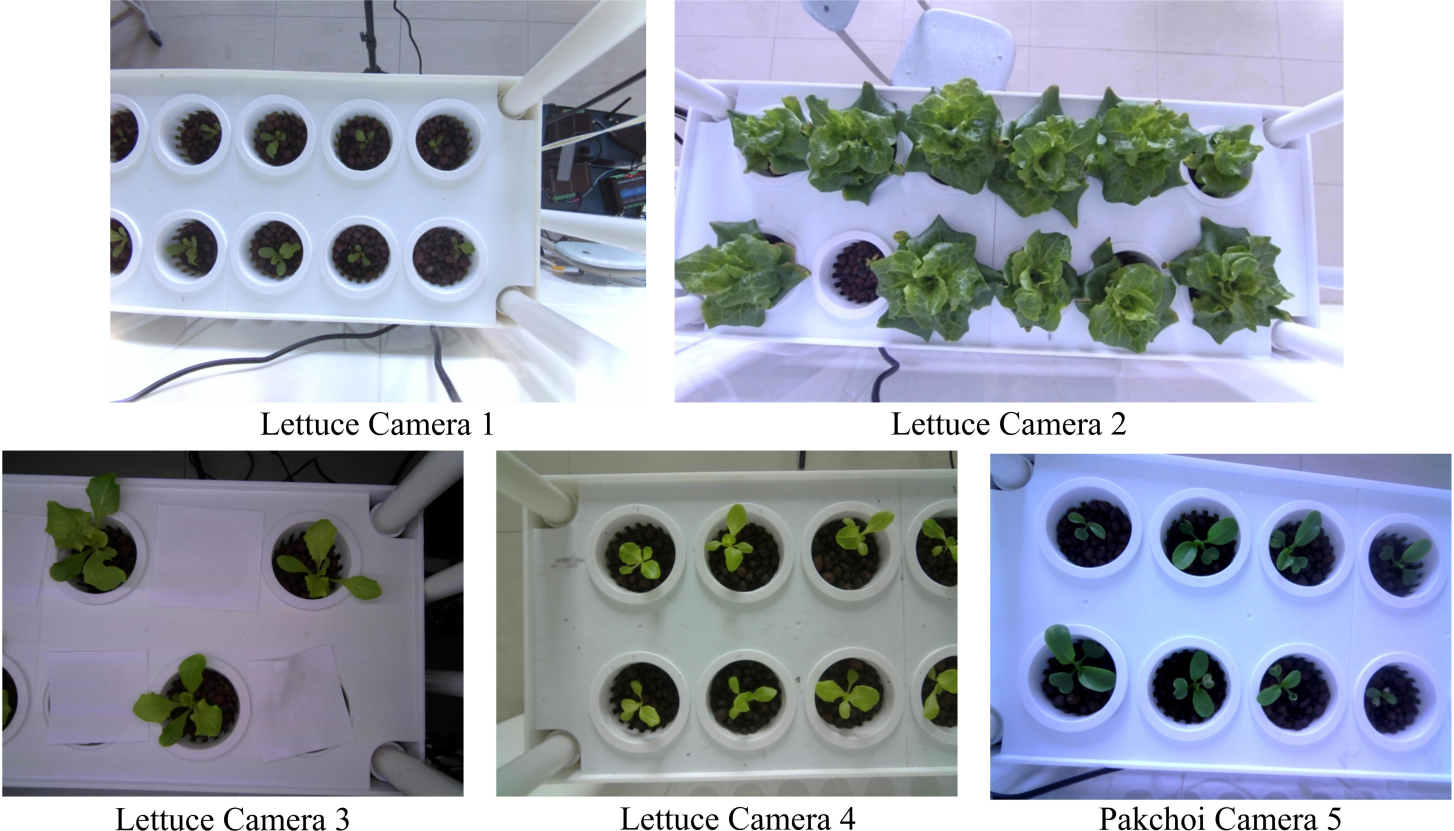
Sample images from HP Dataset.

Lightweight expanded clay aggregate (LECA) was used as the substrate in planting baskets, with one plant planted per basket. Two leafy vegetables, lettuce (Lactuca sativa var. ramosa Hort.) and pakchoi (Brassica chinensis L.), were grown on different layers. A two-year continuous monitoring experiment was conducted using this system in the laboratory at Dali University(25°40’22”N, 100°9’13”E), under controlled conditions: air temperature 15-25°C, hydroponic solution EC 1000 μS/cm (mean), and pH 7.6 (mean), resulting in a collection of over 45,000 images. From these, 203 time-series images representing the main phenological stages of leafy vegetables (germination, seedling, rosette, and heading stages) were selected. These images presented challenges such as complex backgrounds, uneven lighting, and small targets. Image augmentation techniques, including scaling, rotation, and translation, were applied to expand the dataset to 812 images. For segmentation evaluation, all images were manually annotated with leaf boundaries to create a hydroponic plant image dataset (HP Dataset) encompassing the four main phenological stages. The experimental setup consisted of two identical hydroponic racks, each equipped with five cameras (10 cameras total). Following manual screening to eliminate spatial redundancies (e.g., left-right camera pairs within the same tier), five cameras with distinct observational perspectives were selected for time-series data collection—Cameras 1 & 2 (12 MP resolution, 130° diagonal FOV) and Cameras 3–5 (5 MP resolution, 90° diagonal FOV). The numerical designations (1–5) serve as dataset identifiers and do not reflect physical camera placement. Representative images of the two leafy vegetable species are presented in **Figure 2**, with dataset distribution metrics detailed in **Table 2**.

**Table 2:** Data distribution of HP Dataset.

DataSet	Lettuce Camera 1	Lettuce Camera 2	Lettuce Camera 3	Lettuce Camera 4	Pakchoi Camera 5	Total
image count	104	196	116	260	136	812
plant count	1040	2352	396	1820	544	6152

### 2.2 Segmentation with SAM

#### 2.2.1 Overview

**Figure 3:**
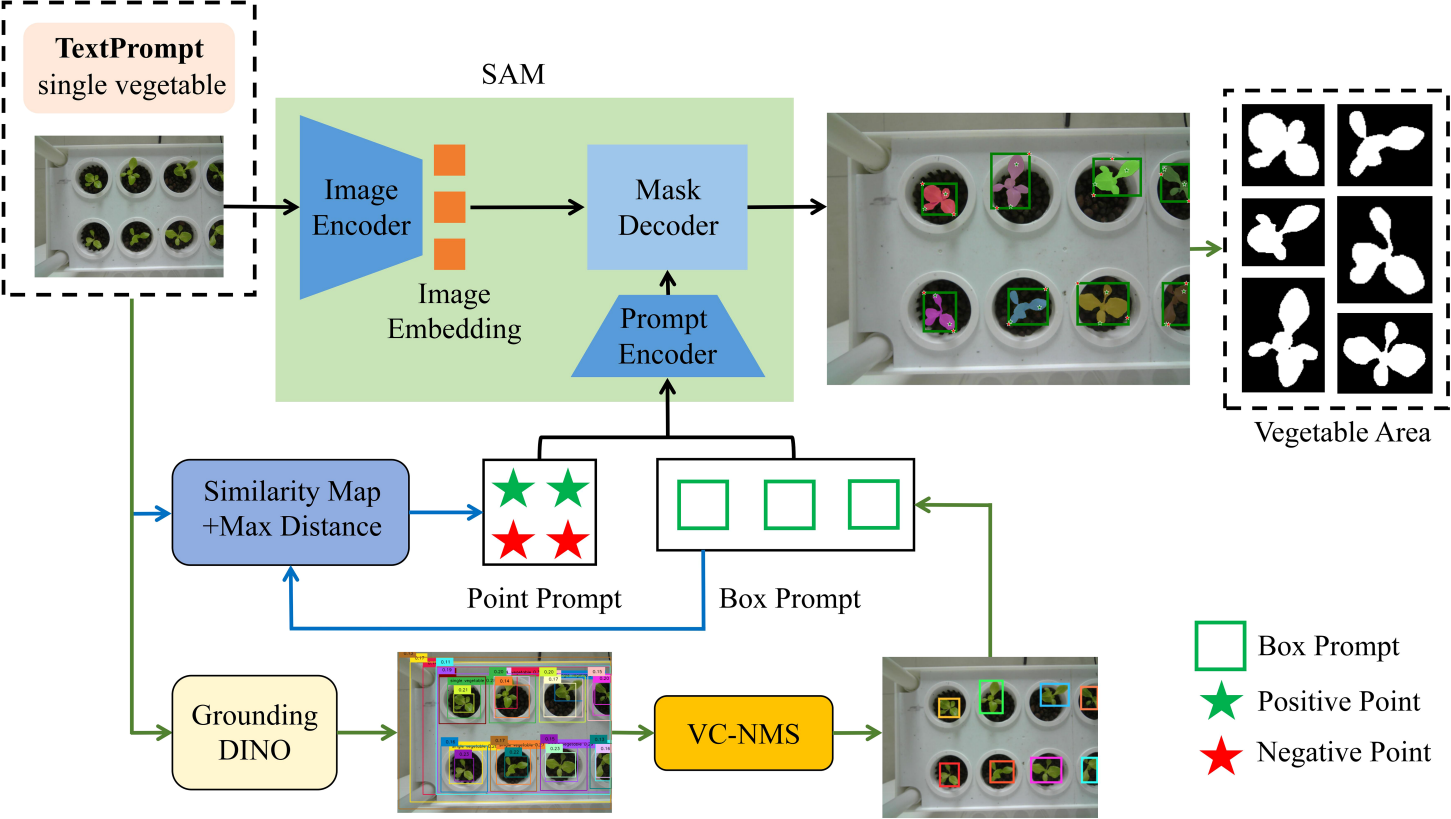
Overview of the automatic segmentation framework. The framework integrates Grounding DINO with VC-NMS to produce box prompts. Point prompts are then generated using the similarity maps and max distance criterion within the boxes. Finally, both box and point prompts are input into SAM’s prompt encoder to guide the segmentation process.

This study introduces a zero-shot plant instance segmentation framework based on a vision foundation model (**Figure 3**). The framework integrates multimodal input, including text and image data, and employs the Segment Anything Model (SAM) for hydroponic plant instance segmentation. To address the prevalent challenge of complex lighting conditions in vertical farms, the images underwent automatic preprocessing to enhance green features. To further improve SAM’s segmentation accuracy for unseen plants, an enhanced box and point prompting strategy was developed to generate effective box and point prompts automatically. Grounding DINO (Liu et al., 2023), a text-prompt-based model, was first used to produce initial candidate bounding boxes for plant instances. These bounding boxes were refined using Vegetation Cover-Aware Non-Maximum Suppression (VC-NMS) to eliminate redundancies and obtain the final bounding boxes, which encapsulate rich semantic information and accurately localize plant instances. Within the final bounding boxes, reference points were extracted using similarity maps, while enhanced points were identified based on a max distance criterion. This refinement process strengthened local feature extraction, improving the quality of point prompts. Finally, the refined bounding boxes and point prompts, serving as spatial structure cues, were integrated into SAM’s segmentation pipeline to direct the model’s attention toward potential plant target regions. By leveraging this synergistic multimodal prompting strategy, the framework significantly enhances SAM’s performance, enabling precise plant instance segmentation in zero-shot scenarios.

#### 2.2.2 Segment anything using SAM

Our core instance segmentation model employs the Segment Anything Model (SAM), which comprises three principal components: image encoder, prompt encoder, and mask decoder. The image encoder transforms input images into high-dimensional feature representations using pre-trained Vision Transformers (ViTs), with available variants including ViT-H (2.4 GB), ViT-L (1.2 GB), and ViT-B (357 MB), where the model size corresponds to pre-training dataset scale. The prompt encoder processes diverse prompt types into image-embedding-compatible features: positional encoding for points/boxes, CLIP text encoder for text prompts, and lightweight convolutional networks for mask inputs. The mask decoder integrates image and prompt embeddings through transformer architecture to generate segmentation masks, typically producing three candidate masks ranked by confidence (only the highest-confidence mask was retained in this study).

Numerous SAM variants have been developed, including MobileSAMv2 (Zhang et al., 2023b), FastSAM (Zhang et al., 2023a), and Lite-SAM (Fu et al., 2024). These implementations employ model compression techniques to enhance inference speed at the cost of segmentation accuracy reduction relative to the original SAM. Another category of domain-specific variants (Zhang et al., 2023c; Li et al., 2023) requires either fine-tuning with annotated datasets or insertion of domain-specific adapters—both of which are incompatible with our zero-shot segmentation objectives. Therefore, we employed the original SAM architecture (Kirillov et al., 2023) with ViT-H image encoder to validate our framework. Our modular implementation maintains low coupling with SAM’s core architecture, enabling seamless integration with future variants while preserving the current pipeline’s functionality.

#### 2.2.3 Box prompt generation using Grounding DINO and VC-NMS

**Figure 4:**
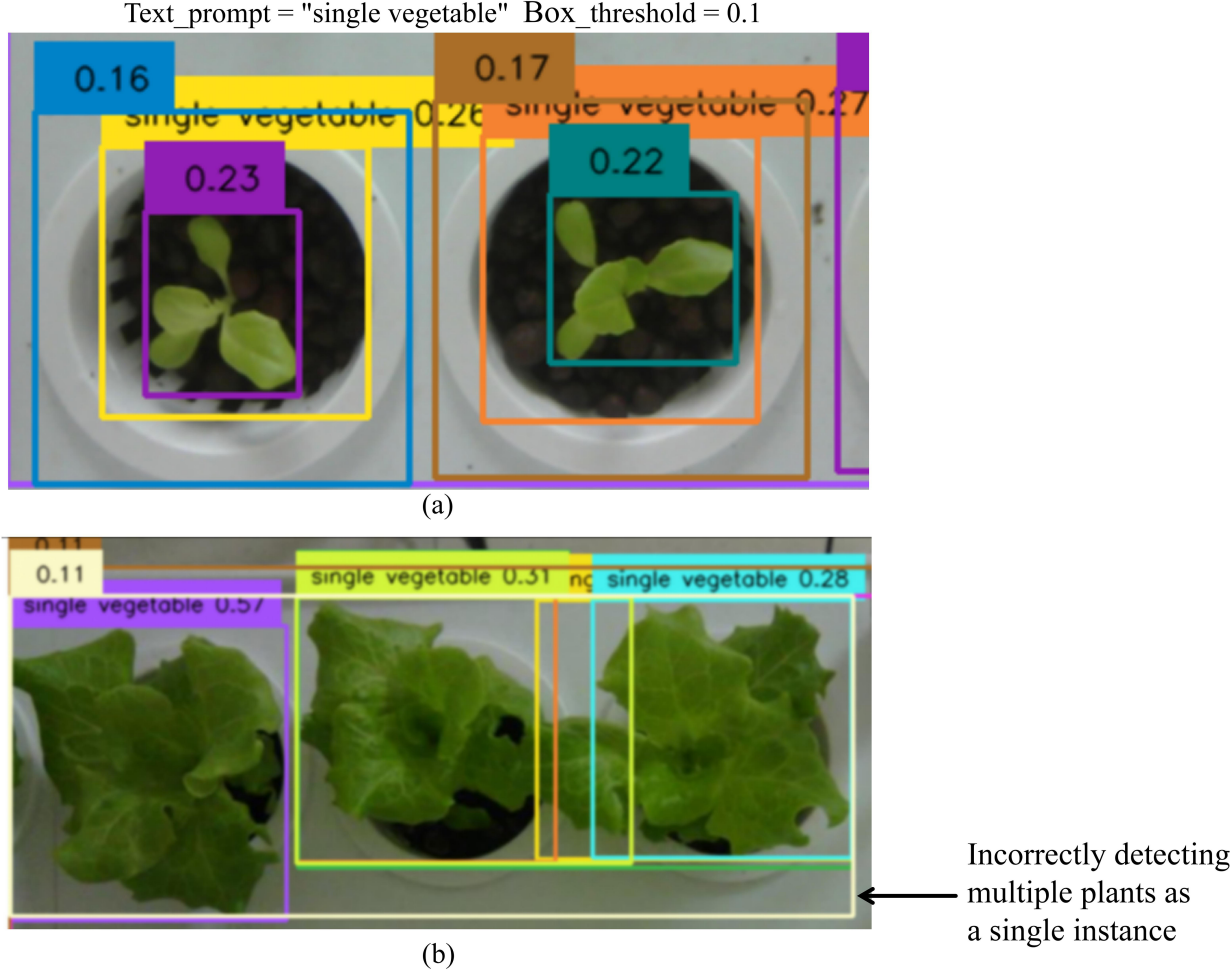
Bounding box generation results from Grounding DINO. Densely overlapping boxes denote candidate regions for the “single vegetable” text prompt. The numbers on these boxes represent confidence scores, which indicate the model's certainty that the box contains a relevant object. However, although a box_threshold of 0.1 is set to filter out low-confidence detections (scores < 0.1), it still leaves a large number of redundant boxes. **(a)** A single plant is enclosed by multiple predicted boxes or partially overlapped. **(b)** Multiple plants are erroneously detected as a single plant.

We use an open-set object detector, called Grounding DINO, by marrying Transformer-based detector DINO with grounded pre-training, which can detect arbitrary objects with human inputs such as category names or referring expressions (Liu et al., 2023). For the backbone network, we select Swin-B due to its larger pretraining dataset and greater parameter capacity. Given an (Image, Text) input pair, Grounding DINO generates multiple detection candidates, each consisting of a bounding box with its corresponding confidence score and associated text phrase. When using Grounding DINO for plant detection, selecting appropriate text prompts can effectively identify plant instances. However, the model frequently generates multiple overlapping bounding boxes (**Figure 4**). This issue becomes particularly evident in complex agricultural images with densely planted plants. In such scenarios, some bounding boxes are nested, where a single object is enclosed by multiple predicted boxes or partially overlapped (**Figure 4a**). Moreover, multiple plants may erroneously be detected as a single instance (**Figure 4b**). We then apply box_threshold filtering to remove all detection boxes whose confidence scores fall below this predetermined threshold value. Increasing the box_threshold values can help filter out low-confidence boxes or those less relevant to the text prompts. However, this approach introduces a tradeoff: reducing these thresholds may lead to missed detections of true targets. Specifically, bounding boxes with confidence scores slightly below the thresholds—despite accurately representing plant locations—might also be excluded.

In object detection tasks, standard Non-Maximum Suppression (NMS) (Neubeck and Van Gool, 2006) and its improved variant, Soft-NMS (Bodla et al., 2017), are widely adopted to reduce redundancy in predicted bounding boxes (Jiang et al., 2023). These algorithms work by selecting the bounding box with the highest confidence score as the reference and suppressing all other boxes with an Intersection over Union (IoU) exceeding a predefined threshold. Their primary objective is to suppress low-confidence bounding boxes that overlap significantly with the reference box. Nevertheless, Grounding DINO’s plant detection often generates a considerable number of redundant nested boxes with similar confidence scores (**Figure 4**). As a result, methods that rely solely on confidence scores and IoU, such as standard NMS and Soft-NMS, are insufficient to effectively remove these redundant bounding boxes.

To overcome the limitations of traditional NMS in Grounding DINO’s plant detection, this study introduces an improved NMS algorithm, Vegetation Cover-Aware NMS (VC-NMS), which is specifically designed to accurately select bounding boxes that encapsulate a single, complete plant instance. VC-NMS prioritizes bounding boxes with higher vegetation cover indices and fewer overlapping boxes, thereby enhancing the precision and reliability of plant detection in complex agricultural scenarios. The algorithm employs the following strategies:

1. Prioritizing bounding boxes with the largest aggregated vegetation cover: We employ a Gaussian decay function based on comprehensive Normalized Cover Green Index (NCGI) values to penalize overlapping boxes, the algorithm prioritizes the box with the highest NCGI. This strategy ensures that the selected bounding box retains the maximal completeness of the enclosed plant instance.
2. Overlapping box suppression mechanism: For large bounding boxes enclosing multiple plants, the algorithm compares the overlap counts of the large box and its nested smaller boxes. If the large box has more overlapping boxes than the smaller one, a linearly decaying penalty function, inversely proportional to the overlap count, is applied to reduce its weight. This mechanism penalizes large boxes with high vegetation cover indices that enclose multiple plants, ensuring accurate retention of individual plant instances.

**Figure 5:**
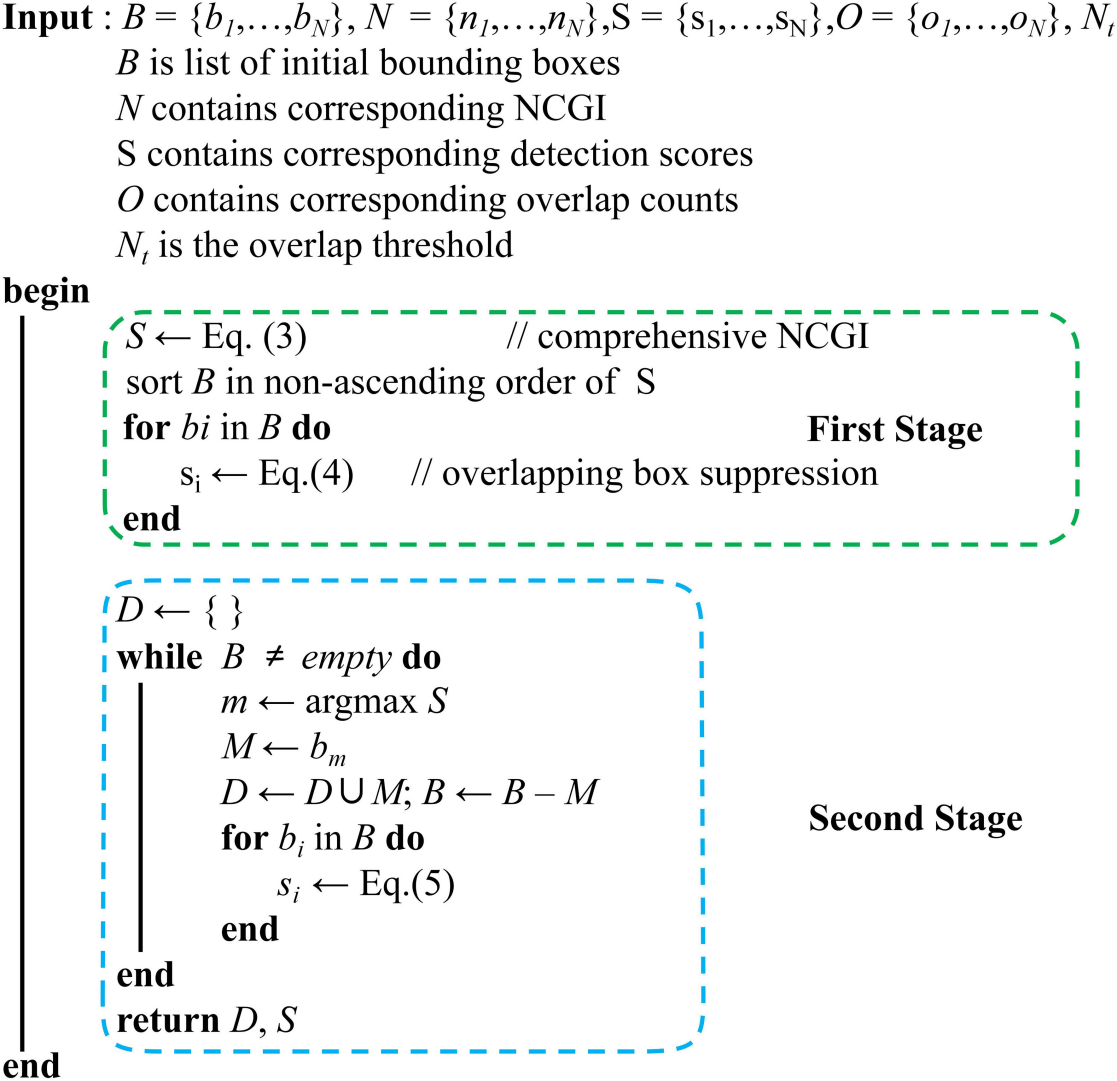
The pseudo code of VC-NMS. Our algorithm consists of two stages: the first stage calculates the integrated NCGI and applies the overlapping box suppression method, while the second stage implements the RIoU-based Gaussian penalty function.

As shown in **Figure 5**, our algorithm starts with a list of bounding boxes B with scores S from Grounding DINO, and calculates corresponding NCGI N and overlap counts O for B. The algorithm has two stages: the first stage updates bounding box scores by calculating comprehensive NCGI and using the overlapping box suppression method, and the second stage selects the detection with maximum score M, removes it from set B and appends it to final detections D. Then it applies the RIoU-based Gaussian penalty function to remaining bounding boxes. Implementation details follow.

The standard Intersection over Union (IoU) metric is insufficient for effectively detecting nested smaller boxes within larger ones, especially when the area of the larger box is significantly greater than that of the smaller box. In such cases, the IoU value may fall below the threshold, failing to capture the nested relationship. In this study, we adopt the Relative IoU (RIoU), which calculates the ratio of the intersection area between two bounding boxes to the total area of the smaller bounding box. Compared to standard IoU, RIoU emphasizes the extent to which the smaller box is covered by the larger box, making it particularly suitable for applications where preserving small targets is critical. This approach helps prevent smaller targets from being overlooked or erroneously suppressed due to the presence of larger targets, as expressed in Equation 1.


(1) $$\text{RIoU}=\frac{A\cap B}{min(A,B)}$$

The confidence scores of plant bounding boxes detected by Grounding DINO are influenced by factors such as plant type, size, lighting, and background, resulting in uncertainty and complicating the selection of the optimal bounding box based solely on confidence. To address this issue, we propose the Normalized Cover Green Index (NCGI) as a confidence metric for plant detection boxes. The NCGI is derived from the Excess Green (EXG) index and is computed by first applying Otsu’s thresholding to binarize the image, followed by calculating the total green pixel count within each bounding box (denoted as NCGI_raw). These raw green pixel counts are then normalized across all overlapping bounding boxes. The NCGI for the i-th bounding box is defined in Equation 2.


(2) $$\text{NCGI}({\text{box}}_{i})=\frac{{\text{NCGI}}_{raw}(box_{i})}{max_{k\in \text{overlap}({\text{box}}_{i})}(NCGI_{raw}({\text{box}}_{k}))}$$

Here, box_*i*_ represents the i-th bounding box for which the NCGI value is being calculated, and *k*∈overlap(box_*i*_) denotes the set of bounding boxes overlapping with box_*i*_.

Standard NMS uses the confidence score of bounding boxes as the basis for suppression. Considering the specific characteristics of plant detection, this study combines the Normalized Cover Green Index (NCGI) with the confidence score to compute a composite NCGI value, which serves as the bounding box score, as defined in Equation 3. However, Grounding DINO’s plant detection often generates a large number of bounding boxes that enclose multiple plants, resulting in excessively high composite scores for these boxes. To address this issue, bounding boxes with high composite scores and a large number of overlapping boxes are penalized by reducing their scores. This suppression mechanism effectively reduces the influence of large boxes enclosing multiple plants.

To balance the dual objectives of prioritizing bounding boxes with the highest composite NCGI values and suppressing boxes with a large number of overlaps, VC-NMS employs a two-stage algorithm. In the first stage, the composite NCGI values, derived from the NCGI value and the confidence score, is assigned as the score for each box. Additionally, boxes with a high number of overlaps are penalized by reducing their scores, with larger penalties applied to boxes enclosing multiple overlapping instances. Let *B* represent the set of *N* bounding boxes detected by Grounding DINO. These boxes are sorted in non-ascending order based on their composite index values. The score *s*_*i*_ for each box *b*_*i*_ in *B* is calculated as shown in Equation 4.


(3) $$s_{i}=\beta \cdot {\text{NCGI}}_{i}+(1-\beta )\cdot s_{i}$$

(4) $$s_{i}=\{\begin{array}{c} s_{i},\text{RIoU}(b_{i},b_{j})<Nt,i\in [1,N]\text{ and }j\in (i,N]\\ (1-{\text{oven}}_{i}\text{/maxoven})\cdot s_{i},\text{RIoU}(b_{i},b_{j})\;\geq \;N_{t}{\text{ and oven}}_{i}\;\geq {\text{ oven}}_{j} \end{array}$$

Here, NCGIi represents the cover green index of box *b_i_*, and RIoU(*b_i_,b_j_*) denotes the relative Intersection over Union (RIoU) between *b_i_* and *b_j_*. Overlap threshold *N_t_* =0.7 ensures that only highly overlapping boxes receive overlap penalty during the first processing stage. The parameter *β* serves as the weight of the NCGI component, with 0 ≤ *β* ≤ 1. This parameter can be adjusted in practical applications based on the degree of plant density and occlusion. During fully automated batch testing, *β* is set to 0.7 to balance the vegetation cover index and confidence score. The overlap index, oven*_i_*, represents the number of overlapping boxes for box *b_i_*, while max_oven denotes the highest overlap index among all boxes. A linear attenuation function is applied to penalize *s_i_*, where a higher overlap index results in a greater penalty.

The second stage of VC-NMS is similar to Soft-NMS, with the objective of suppressing the scores of overlapping bounding boxes and removing redundant boxes. Unlike Soft-NMS, VC-NMS replaces the standard Intersection over Union (IoU) with the Relative Intersection over Union (RIoU). Let *M* represent the bounding box with the highest score, and let *B*={*b_i_* |1≤*i*≤*N*} denote boxes not in the final set D, the score of *b_i_* is reduced using a decay function *f*. The decay function is defined in Equation 5.


(5) $$s_{i}=s_{i}\cdot f(\text{RIOU}(M,b_{i}),\forall bi\notin D$$

Here, *f*(RIoU(*M*,*B_i_*)) represents a weight-based decay function designed to reduce the scores of overlapping bounding boxes. In Grounding DINO, multiple nested boxes are often detected for the same plant, resulting in very high RIoU values between these boxes. Such boxes should receive a significant penalty. Conversely, overlapping boxes corresponding to adjacent plants typically have lower RIoU values and should receive lighter penalties. Considering these factors, we adopt the Gaussian penalty function from Soft-NMS. The decay function is defined in Equation 6. Unlike traditional NMS with hard thresholds, the Gaussian function provides smooth suppression, avoiding abrupt score discontinuities. The continuous penalty function has no penalty when there is no overlap and very high penalty at a high overlap.


(6) $$f(\text{RIoU}(M,b_{i}))=e^{-\;\frac{\text{RIoU}{\left(M,b_{i}\right)}^{2}}{\sigma }}$$

#### 2.2.4 Point prompt generation using similarity maps and max distance criterion

**Figure 6:**
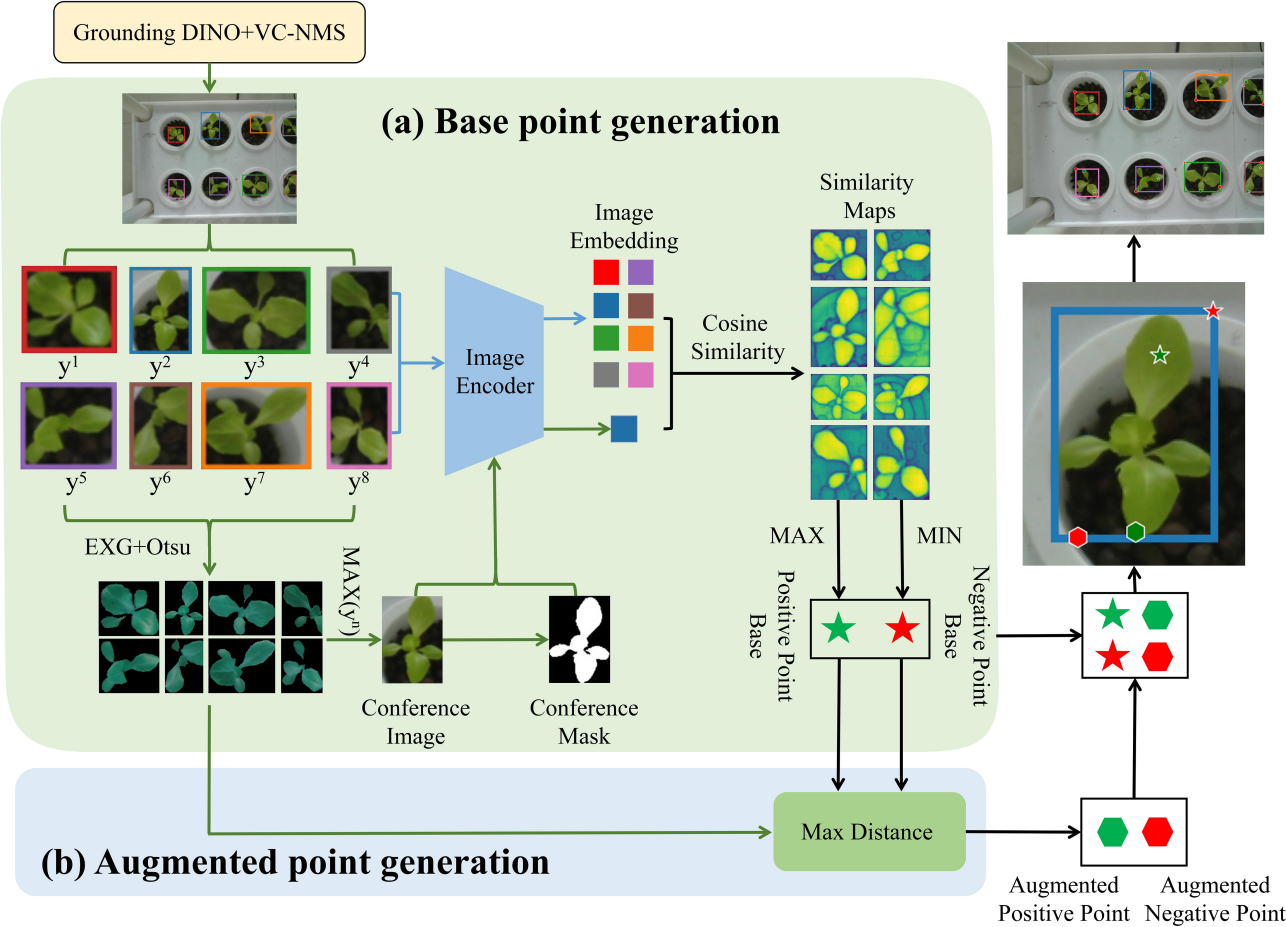
Point prompt augmentation flowchart. **(a)** Base points are generated using a similarity map, where *y^n^* represents the composite NCGI value of each bounding box region. The points with the highest and lowest similarity values in each similarity map are selected as the base positive and negative points, respectively. **(b)** Enhanced points are generated using the max distance criterion within the foreground and background regions initially identified by the EXG and OTSU method.

The point prompt augmentation method consists of two steps. First, base points are generated using similarity maps. Then, enhanced points are determined using the max distance criterion (**Figure 6**).

1. Generation of Base Points Using Similarity Maps.

**Figure 7:**
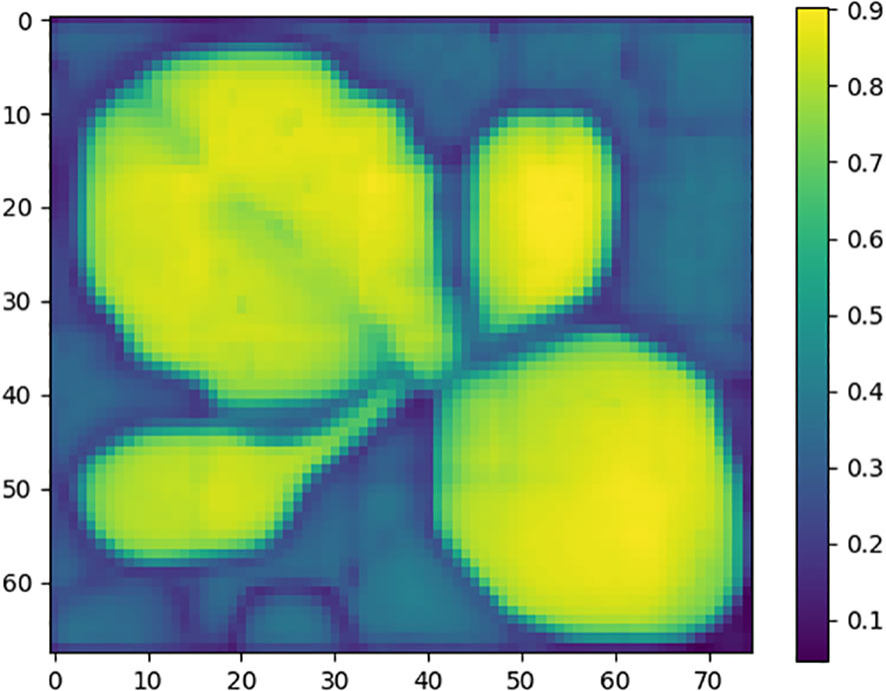
Similarity map. The figure illustrates the matching relationship between the target plant regions and the reference plant region. Different colors represent varying levels of similarity, with brighter colors indicating higher similarity.

First, each bounding box region obtained by Grounding DINO and VC-NMS is assigned a composite NCGI value (*y^n^*). The bounding box with the highest *y^n^* is selected as the reference box, and its image is cropped to serve as the reference image for generating point prompts. To enhance the green features of the plant, the Excess Green (EXG) algorithm is applied to the reference image, followed by the OTSU method to segment the enhanced image into foreground and background regions. The foreground region is then used to generate a reference mask. After obtaining the reference image and reference mask, the SAM image encoder is used to extract features from both. Subsequently, features are extracted from all bounding box region images. The image embedding of the reference image is then compared with the embeddings of all bounding box regions by calculating the cosine similarity, generating a similarity map, as shown in **Figure 7**. In the similarity map, the point with the highest similarity value is selected as the positive base point, while the point with the lowest similarity value is selected as the negative base point for each bounding box region.

(2) Generation of Enhanced Points Based on Base Points Using the Maximum Distance Criterion.

We referred to the study by Dai et al. (2023) on SAM point prompt augmentation. For each target region, the EXG algorithm was applied to enhance green features, followed by the OTSU method to segment the image into preliminary foreground and background regions. Enhanced points were then generated within these regions using the base positive and negative points obtained in the first step. Specifically, the Euclidean distance was calculated, and the farthest point from the base point was selected as the enhanced point.

### 2.3 Evaluation metrics

To comprehensively evaluate the performance of bounding box detection and instance segmentation, we employed several widely used metrics from object detection and image segmentation. These metrics include Intersection over Union (IoU), Precision (P), Recall (R), Average Precision (AP), Dice Similarity Coefficient (Dice), Expected Calibration Error (ECE) (Guo et al., 2017), Structure Measure (Sm) (Fan et al., 2017), and Weighted F-measure (wFm) (Margolin et al., 2014). The definitions and formulations of these metrics are provided in Equations 7-13.


(7) $$P=\frac{TP}{TP+FP}$$

(8) $$R=\frac{TP}{TP+FN}$$

(9) $$AP=\int_{0}^{1}PdR$$

(10) $$Dice=\frac{2\cdot |A\cap B|}{\left|A|+|B\right|}$$

(11) $$ECE=\sum_{m=1}^{M}\frac{\left|B_{m}\right|}{n}|acc(B_{m})-conf(B_{m})|$$

(12) $$Sm=\alpha \cdot S_{\text{object}}+(1-\alpha )\cdot S_{\text{region}}$$

(13) $$F_{\beta }^{\omega }=(1+\beta^{2})\frac{{\text{Precision}}^{\omega }\cdot {\text{Recall}}^{\omega }}{\beta^{2}\cdot {\text{Precision}}^{\omega }+{\text{Recall}}^{\omega }}$$

Intersection over Union (IoU) quantifies the overlap between predicted and ground truth segmentation masks, with higher values indicating better alignment. True Positive (TP) refers to the number of correctly predicted bounding boxes or masks where the Intersection over Union (IoU) between the predicted and ground truth boxes or masks meets or exceeds the IoU threshold. Predictions falling below this threshold are classified as False Positives (FP), while False Negatives (FN) represent the number of ground truth bounding boxes or masks missed by the predictions. Precision (P) (Equation 7) quantifies the proportion of true positive predictions among all positive predictions. Higher precision indicates that the model makes fewer false positive predictions, meaning that when it predicts an object, it is more likely to be correct. Recall (R) (Equation 8) measures the proportion of true positive predictions among all ground truth instances. Higher recall indicates that the model is able to identify most of the true positive instances, meaning fewer false negatives. In Equation 9, Average Precision (AP) is defined as the area under the Precision-Recall (P-R) curve computed at different IoU thresholds. This study employs AP@0.5 and AP@0.5:0.95, where AP@0.5 represents the average precision at an IoU threshold of 0.5, and AP@0.5:0.95 represents the mean average precision calculated across IoU thresholds ranging from 0.5 to 0.95, with intervals of 0.05. The Dice Similarity Coefficient (Dice) (Equation 10) measures the degree of overlap between predicted and ground truth regions. It ranges from 0 to 1, with values closer to 1 indicating better segmentation performance. Expected Calibration Error (ECE) (Equation 11) evaluates the consistency between predicted probabilities and observed outcomes, providing a measure of calibration quality in probabilistic models. Structure Measure (Sm) (Equation 12) assesses the structural similarity or quality between two images. Unlike pixel-level metrics, Sm emphasizes structural information such as edges, textures, and shapes. Setting *α*=0.5 balances the weights between object-aware and region-aware similarities. Weighted F-measure (wFm) (Equation 13) extends the traditional F-measure by incorporating the varying importance of different regions or objects within an image. This modification aligns the evaluation results with the requirements of specific application scenarios.

## 3 Experimental results

### 3.1 Performance evaluation of bounding box prompts

**Figure 8:**
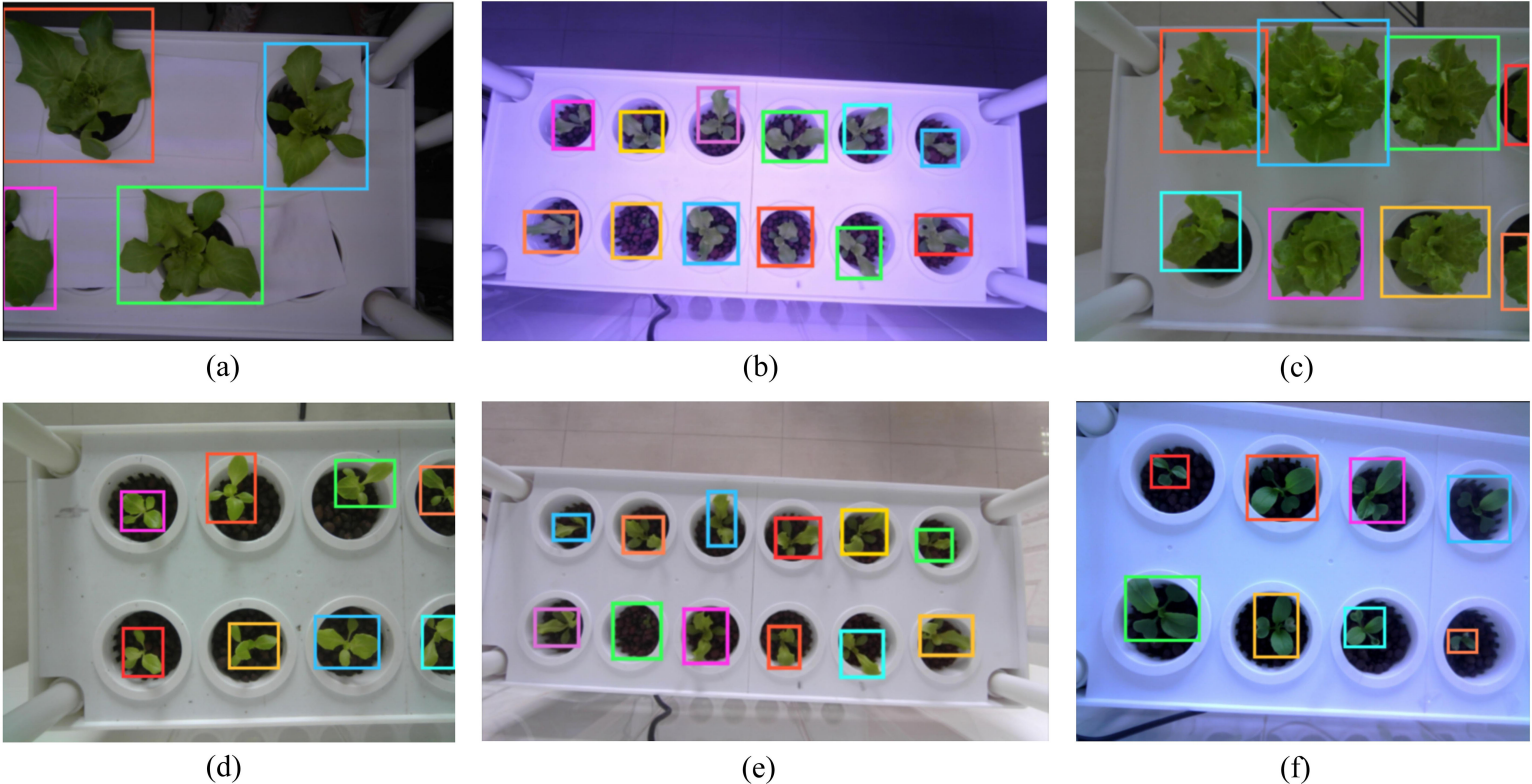
Results of individual plant detection using Grounding DINO combined with VC-NMS.

Experiments on zero-shot single-plant detection using Grounding DINO were conducted on the hydroponic plant dataset (HP Dataset). The text_prompt for Grounding DINO was set as “single vegetable” with the Box Threshold values set to 0.1. To remove redundant boxes, the proposed VC-NMS algorithm was applied. Examples of detected single plants are shown in **Figure 8**. The model successfully identified individual plants across various scenarios, including significant variations in plant scale and viewing angles (**Figure 8e**), plant types (**Figure 8f**), planting methods (**Figure 8a**), and lighting conditions (**Figure 8b**).

To validate the performance of our proposed VC-NMS in removing redundant bounding boxes generated by Grounding DINO, we compared its detection accuracy with that of Greedy-NMS (G-NMS), Soft-NMS with a linear penalty function (S-NMS(L)), and Soft-NMS with a Gaussian penalty function (S-NMS(G)). The experimental results, shown in **Table 3**, demonstrate that our algorithm achieves significant performance improvements, outperforming the others across all four metrics. Specifically, VC-NMS achieved the highest AP@0.5 (0.699) and the highest Recall@10 (0.659). These results indicate that VC-NMS effectively leverages the color information of hydroponic plants, allowing it to better remove redundant bounding boxes generated by Grounding DINO in complex environments. Quantitative analysis of AP across varying target scales reveals that the Grounding DINO with NMS framework exhibits a fundamental limitation in detecting small objects (defined as targets with diagonal lengths <32 pixels). This performance constraint primarily originates from the box confidence threshold (box_threshold =0.1 in our implementation) – small objects inherently generate low-confidence predictions (typically below the 0.1 threshold), resulting in their systematic exclusion during the initial filtering phase. However, arbitrarily reducing this parameter is impractical, as it would produce excessive non-overlapping candidate bounding boxes that exceed the suppression capacity of conventional NMS algorithms due to their spatially dispersed distribution.

**Table 3:** Performance evaluation of grounding DINO with VC-NMS.

Method	AP@0.5	AP@0.75	AP@0.9	AP 0.5:0.95	Recall 10	Recall 100	AP Small	AP Medium	AP Large
G-NMS	0.522	0.338	0.230	0.350	0.515	0.537	0.091	0.446	0.723
S-NMS (L)	0.581	0.379	0.261	0.393	0.537	0.563	0.091	0.497	0.782
S-NMS (G)	0.542	0.345	0.233	0.360	0.494	0.506	**0.100**	0.480	0.732
VC-NMS	**0.699**	**0.559**	**0.407**	**0.541**	**0.659**	**0.675**	0.091	**0.658**	**0.770**

S-NMS (L) indicates that Soft-NMS uses a linear penalty function, while S-NMS (G) indicates that Soft-NMS uses a Gaussian penalty function.

The optimal performance metrics are shown in bold.

**Figure 9:**
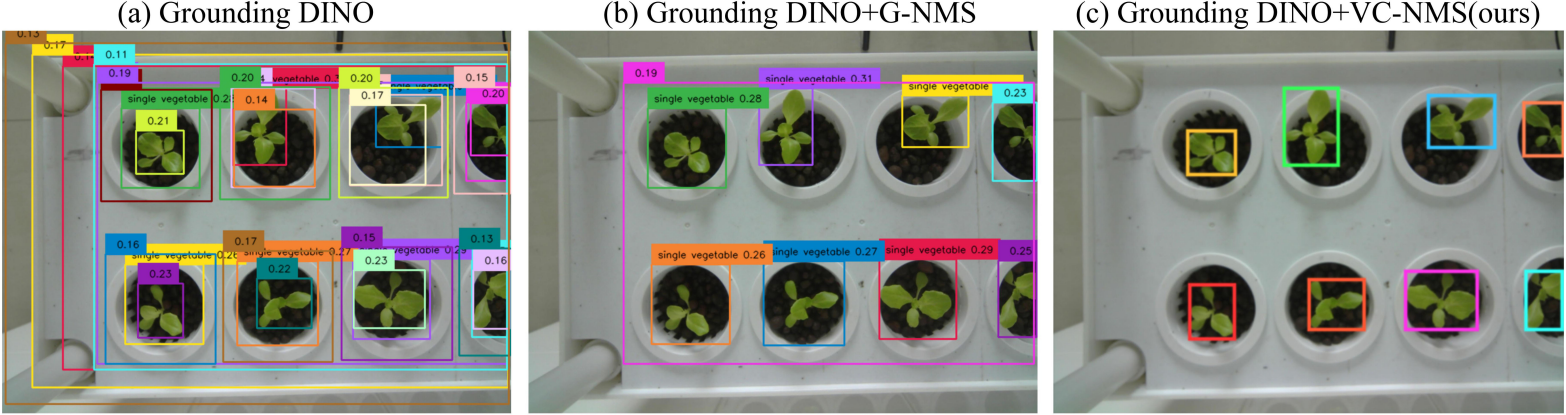
Qualitative results of redundant bounding box removal using Greedy-NMS (G-NMS) and VC-NMS in Grounding DINO. **(a)** Initial candidate boxes generated by Grounding DINO (confidence scores shown at top-left), where boxes below text_threshold = 0.1 are filtered. **(b)** Results after G-NMS processing. **(c)** Results after VC-NMS processing, demonstrating superior box selection.

**Figure 9** presents a qualitative comparison of redundant bounding box removal using Greedy-NMS (G-NMS) and VC-NMS. Larger boxes detected by Grounding DINO often exhibit higher confidence scores due to its detection mechanism (**Figure 9a**). However, these larger boxes often include significant redundant areas. G-NMS tends to prioritize these high-confidence large boxes by default (**Figure 9b**), which are suboptimal as prompts for SAM. Oversized boxes can substantially reduce the instance segmentation performance of SAM. In contrast, VC-NMS considers both the composite NCGI value and the number of overlapping boxes. By suppressing large boxes with significant overlaps and prioritizing those with higher composite NCGI values, VC-NMS effectively selects bounding boxes that are complete and free of redundancy (**Figure 9c**). This approach generates more effective prompts for SAM, leading to improved instance segmentation accuracy.

### 3.2 Performance evaluation of point prompts

**Figure 10:**
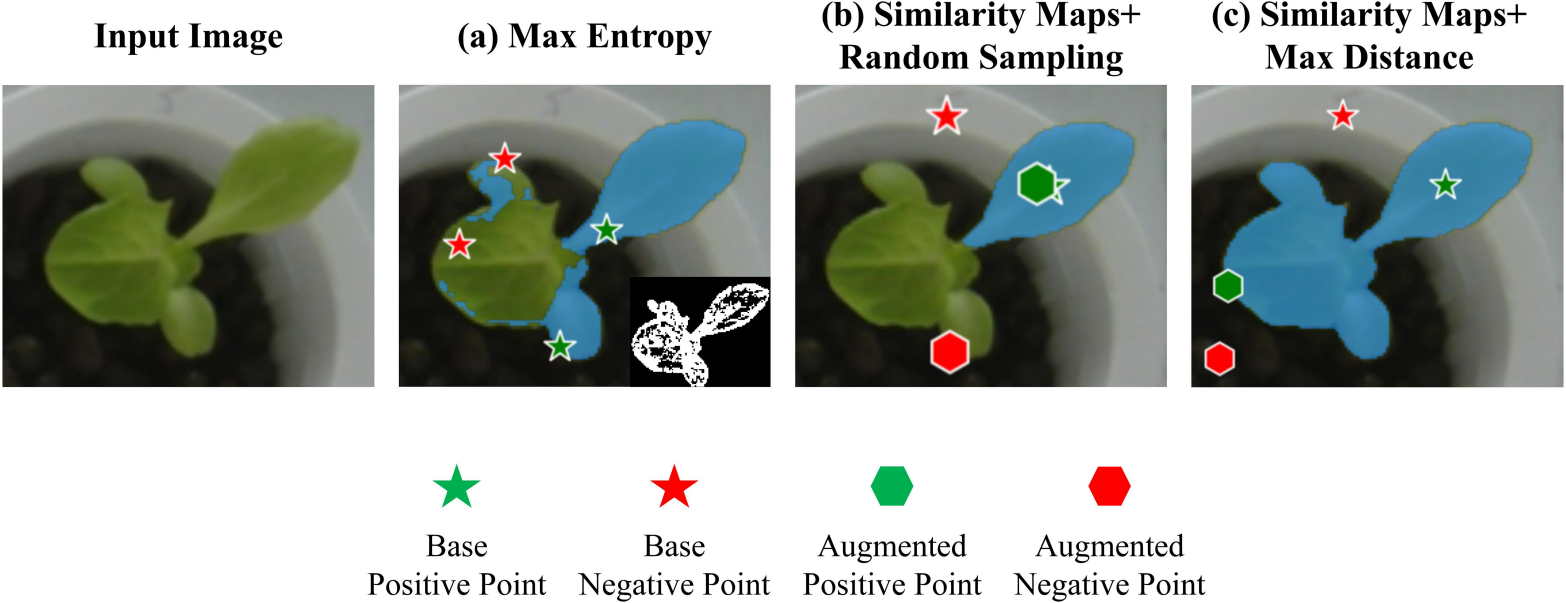
Comparison of the effects of different point augmentation strategies. **(a)** Max Entropy Criterion: The bottom-right corner shows the binarized entropy map. Negative points located within plant regions lead to significant negative impacts on segmentation performance. **(b)** Similarity Maps with Random Sampling: Randomly generated enhanced points tend to cluster within a specific area, reducing the effectiveness of the prompts. **(c)** Similarity Maps with Max Distance: This method effectively utilizes spatial information from the image and generates accurate point prompts.

To validate the effectiveness of the proposed point prompt augmentation strategy based on the similarity maps and max distance criterion, comparative experiments were conducted using max entropy criterion (Kapur, 1989) and similarity maps with random sampling as baseline methods. In the max entropy criterion, the entropy value of every pixel in the image is calculated, and high-entropy regions are separated using a predefined threshold to generate a green entropy map. Positive prompt points are then randomly sampled from the green entropy maps within the bounding boxes generated by Grounding DINO and VC-NMS, while negative points are randomly selected outside the green entropy map. For similarity maps with random sampling, base positive and negative points are first determined by calculating similarity values. Then, additional points are randomly sampled from the foreground and background regions preliminarily segmented using EXG and OTSU. **Figure 10** shows a comparison of prompt points generated using different augmentation strategies. The max entropy criterion suffers from discontinuous entropy values, leading to the selection of negative points within low-entropy regions of the plant, which can severely degrade segmentation performance. On the other hand, similarity maps with random sampling, due to their inherent randomness, may generate overly dense distributions of prompt points, reducing the amount of effective information provided to SAM and slightly underperforming the max distance criterion. Its instability further limits its practical applicability. In contrast, the proposed similarity maps with max distance criterion generate stable and effective prompts, enabling SAM to better focus on the entire plant region. This results in superior segmentation performance compared to the baseline methods.

To assess how the number of positive and negative prompt points influences SAM’s instance segmentation performance, we conducted a series of comprehensive experiments. Experimental results presented in **Table 4** show that the highest Dice score is achieved when the number of positive and negative prompt points is set to two. Both excessive and insufficient prompt points lead to a decline in SAM’s instance segmentation performance. Hence, determining the optimal number of prompt points is essential for maximizing SAM’s instance segmentation performance.

**Table 4:** Comparison of the performance impact of different numbers of point prompts (unit: Dice score).

	1Positive	2Positive	3Positive	4Positive	5Positive
1Negative	0.697	0.709	0.704	0.695	0.697
2Negative	0.699	**0.712**	0.709	0.703	0.697
3Negative	0.698	0.708	0.705	0.698	0.689
4Negative	0.695	0.704	0.701	0.692	0.684
5Negative	0.693	0.700	0.692	0.677	0.680

The optimal performance metric is shown in bold.

### 3.3 Performance evaluation in the zero-shot instance segmentation task

#### 3.3.1 Comparison with the baseline approaches

##### 3.3.1.1 Quantitative results on the HP dataset

The proposed method was quantitatively evaluated against zero-shot baseline methods based on SAM, including SAM’s everything mode and Grounded SAM (box_threshold = 0.3), on the HP Dataset. The results are presented in **Table 5**. Across three different backbones, neither SAM’s everything mode nor Grounded SAM showed significant improvements in instance segmentation performance as the parameter size of the image encoder increased. This limitation may stem from their inability to precisely localize plant regions. In contrast, the proposed method achieves superior performance across all evaluated metrics, obtaining an IoU of 0.593, a Dice of 0.712, a Sm of 0.771, a wFm of 0.769, and the lowest ECE of 0.009. Compared to SAM’s everything mode and Grounded SAM, the proposed method achieves significant improvements in instance segmentation performance while retaining the zero-shot framework.

**Table 5:** Performance comparison with zero-shot baseline methods based on SAM.

Method	Backbone	IoU↑	Dice↑	ECE↓	Sm↑	wFm↑
SAM Everything	ViT-B	0.194	0.286	0.326	0.573	0.330
	ViT-L	0.156	0.239	0.600	0.574	0.277
	ViT-H	0.148	0.228	0.667	0.570	0.265
Grounded SAM	ViT-B	0.464	0.584	0.027	0.700	0.634
	ViT-L	0.470	0.578	0.024	0.676	0.625
	ViT-H	0.453	0.565	0.027	0.666	0.614
Ours	ViT-B	**0.576**	**0.697**	**0.011**	**0.758**	**0.755**
	ViT-L	**0.585**	**0.706**	**0.010**	**0.769**	**0.762**
	ViT-H	**0.593**	**0.712**	**0.009**	**0.771**	**0.769**

Optimal results per backbone are shown in bold. The '↑' symbol indicates that higher values are better for this metric, while the '↓' symbol indicates that lower values are better for this metric.

##### 3.3.1.2 Qualitative visual comparisons

**Figure 11:**
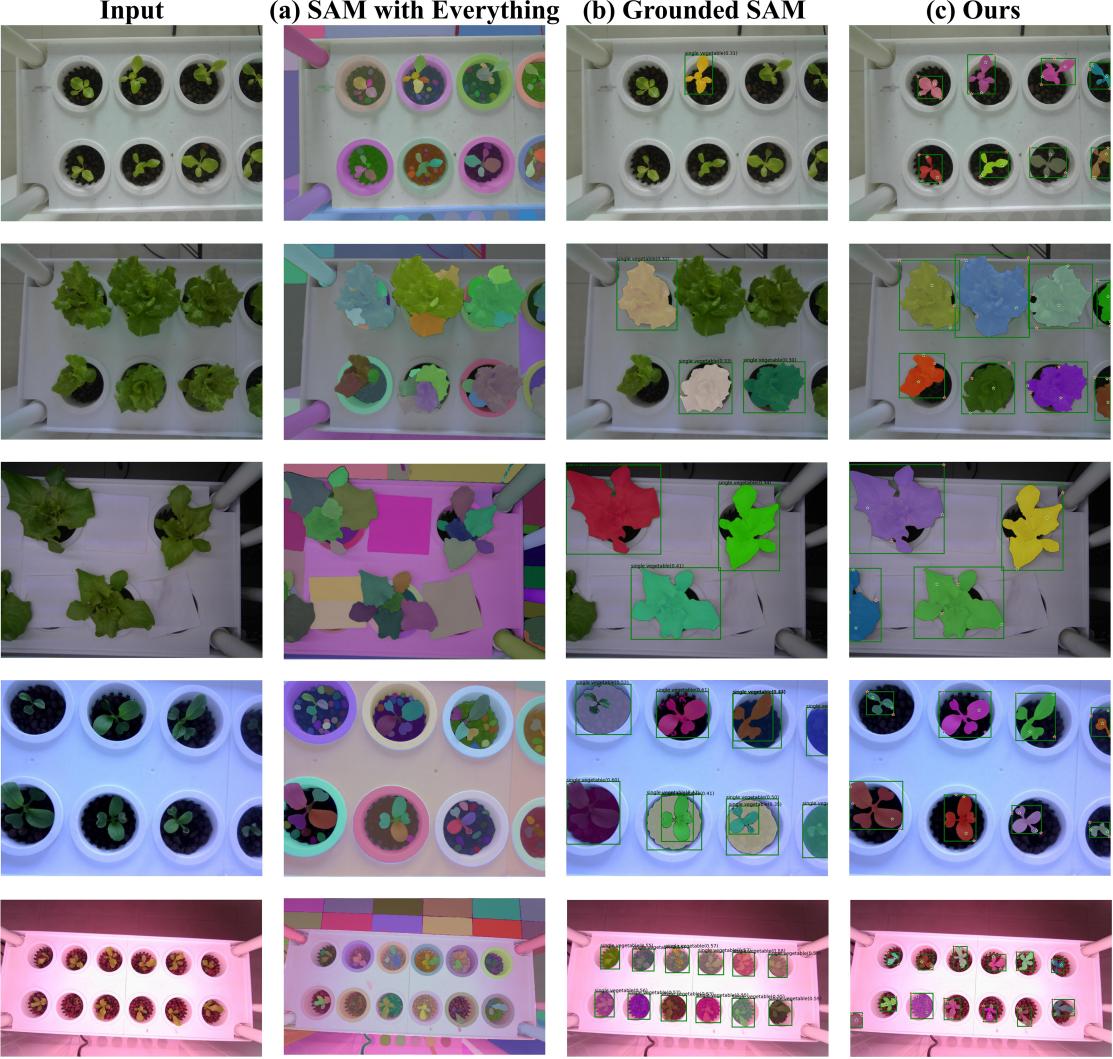
Qualitative segmentation results. Our method is compared with baseline methods. Even under challenging lighting conditions (fifth row), our model exhibits robust instance segmentation performance.

To demonstrate the effectiveness of the proposed prompt augmentation strategy, qualitative comparisons were conducted between our method, SAM’s Everything Mode, and Grounded SAM, as shown in **Figure 11**. Within the same zero-shot segmentation framework, SAM’s Everything Mode generates overly fine-grained segmentation results, frequently splitting a single plant into separate leaves. Additionally, without prompts, it struggles to accurately localize plants and often includes background regions in the segmentation results (**Figure 11a**). Grounded SAM, in contrast, depends solely on the box_threshold and text_threshold parameters from Grounding DINO to filter bounding boxes. When the thresholds are set too low, numerous redundant boxes are generated; when set too high, many plant targets are missed (**Figure 11b**). By contrast, our method achieves the highest IoU of 0.593 and Dice of 0.712 with the ViT-H backbone. Despite the limitations of Grounding DINO’s thresholds in detecting small targets, the incorporation of VC-NMS for removing redundant boxes, along with enhanced positive and negative point prompts, ensures accurate localization of most plant targets. Consequently, our method achieves precise instance segmentation performance (**Figure 11c**).

#### 3.3.2 Ablation study

In this section, we perform ablation studies to verify the effectiveness of the model design. Specifically, we evaluate the performance of using point prompts and box prompts individually and compare their results with the complete model using both prompts, with the ViT-H backbone. As shown in **Table 6**, while both point prompts and box prompts independently achieve good results, it is evident that combining both prompts yields the best performance.

**Table 6:** Ablations for our model.

Model	IoU	Dice
SAM + Point	0.584	0.695
SAM + Box	0.561	0.687
SAM + Box + Point	**0.593**	**0.712**

The optimal performance metrics are shown in bold.

#### 3.3.3 Sensitivity analysis

In the first stage, VC-NMS introduces hyperparameter *β* to balance the weights between NCGI and confidence score in the composite NCGI calculation, as shown in Equation 3. In practice, *β* can be adjusted according to plant growth stages: set lower in early growth phases to emphasize confidence scores, and higher in later phases to enhance NCGI contribution for better segmentation. For batch experiments requiring a fixed *β* value, we conducted sensitivity analysis on the HP dataset, with results shown in **Table 7**. The segmentation performance remained stable with *β* values between 0.4-0.9, achieving optimal results at *β* = 0.7. Therefore, we set *β*=0.7 for all experiments without further dataset-specific tuning.

**Table 7:** Parameter sensitivity analysis for *β* values with ViT-B image encoder architecture.

β	IoU↑	Dice↑	ECE↓	Sm↑	wFm↑
0.1	0.576	0.694	0.016	0.762	0.751
0.2	0.580	0.697	0.015	0.764	0.755
0.3	0.585	0.703	0.013	0.767	0.761
0.4	0.590	0.709	0.010	0.770	0.766
0.5	0.590	0.709	0.010	0.770	0.766
0.6	0.592	0.711	0.010	0.770	0.768
**0.7**	**0.593**	**0.712**	**0.009**	**0.771**	**0.769**
0.8	0.591	0.710	0.009	0.769	0.768
0.9	0.590	0.710	0.009	0.769	0.768

The optimal segmentation performance metrics are shown in bold.

The '↑' symbol indicates that higher values are better for this metric, while the '↓' symbol indicates that lower values are better for this metric.

#### 3.3.4 Zero-shot generalization ability

**Figure 12:**
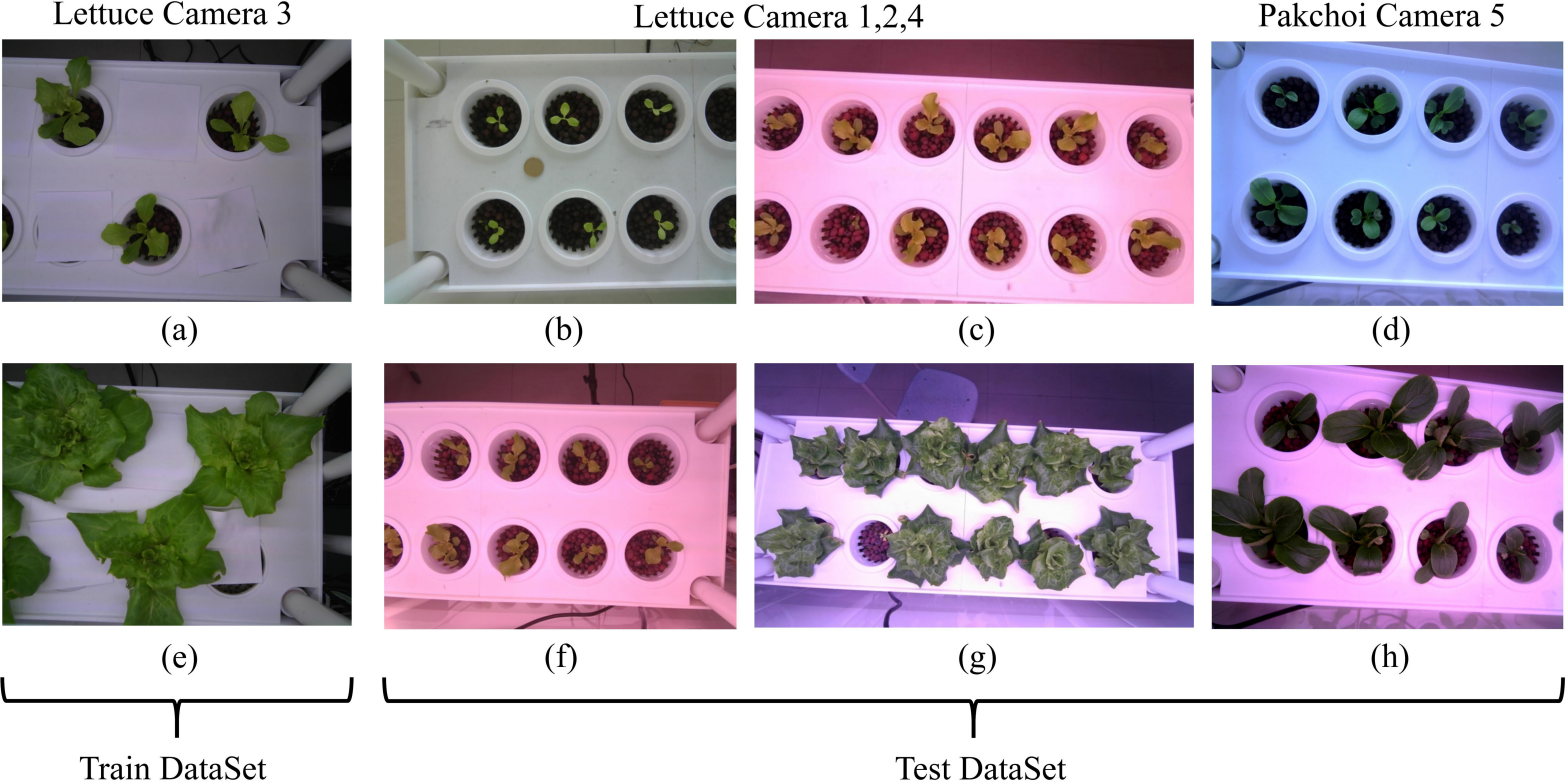
Sample images from the training and test datasets. The test datasets show significant differences from the training dataset in lighting, planting methods, scale, viewing angles, and plant types. **(a, e)** Spaced planting. **(b, d)** Natural lighting effects. **(c, f–h)** Red and purple lighting effects. **(c, f, g)** Larger field of view and scale. **(d, h)** Pakchoi.

To evaluate the zero-shot generalization capability of our model in instance segmentation across different plants and environmental conditions, we conducted comparative experiments using our method (with the ViT-H backbone) and conventional supervised segmentation techniques. Specifically, lettuce images from Camera 3(104 images) were utilized as the training dataset to train SOLOv2 (Wang et al., 2020) and YOLOv11 models. Two independent test sets were used for validation: lettuce images from Cameras 1, 2, and 4(572 images), along with pakchoi images from Camera 5(136 images). These tests assessed the generalization ability of our model and supervised methods across diverse conditions, including variations in lighting, planting methods, scales, viewing angles, and plant types. **Figure 12** shows sample images from the training and test sets, highlighting significant differences in lighting, planting methods, scales, viewing angles, and plant types. **Table 8** provides a performance comparison between our zero-shot model and the supervised segmentation models across each dataset. On the training set, SOLOv2 and YOLOv11 show superior performance, with YOLOv11 achieving the highest IoU (0.768) and the highest Dice (0.862). Notably, our model also performs well on the training set, achieving an IoU (0.714) and an Dice (0.810). However, on the two test sets, the supervised models exhibit significant drops in IoU and Dice, likely caused by substantial differences between the test and training datasets. In contrast, our zero-shot model achieves the best performance on both test sets, demonstrating strong generalization ability and robustness under zero-shot conditions. This capability stems from the fact that both Grounding DINO and SAM were pre-trained on large-scale, diverse datasets, enabling exceptional zero-shot performance in plant localization and precise botanical segmentation. The advantage extends beyond hydroponic systems and can be readily transferred to other green plants through simple text_prompt adaptation.

**Table 8:** Performance comparison between zero-shot and supervised segmentation models.

Model	Lettuce Camera 3		Lettuce Camera 1,2,4		Pakchoi Camera 5	
	IoU	Dice	IoU	Dice	IoU	Dice
SOLO-v2	0.741	0.845	0.412	0.485	0.101	0.145
YOLO-v11	**0.768**	**0.862**	0.500	0.616	0.210	0.300
Ours	0.714	0.810	**0.593**	**0.711**	**0.486**	**0.631**

The optimal performance metrics are shown in bold.

## 4 Discussion

SAM is an interactive instance segmentation model that provides multiple prompting methods, including point, box, mask, and text prompts. Among these, point and box prompts are the most frequently used. Generating and effectively utilizing high-quality point and box prompts are essential for achieving optimal instance segmentation results.

### 4.1 How box prompts influence segmentation performance

**Figure 13:**
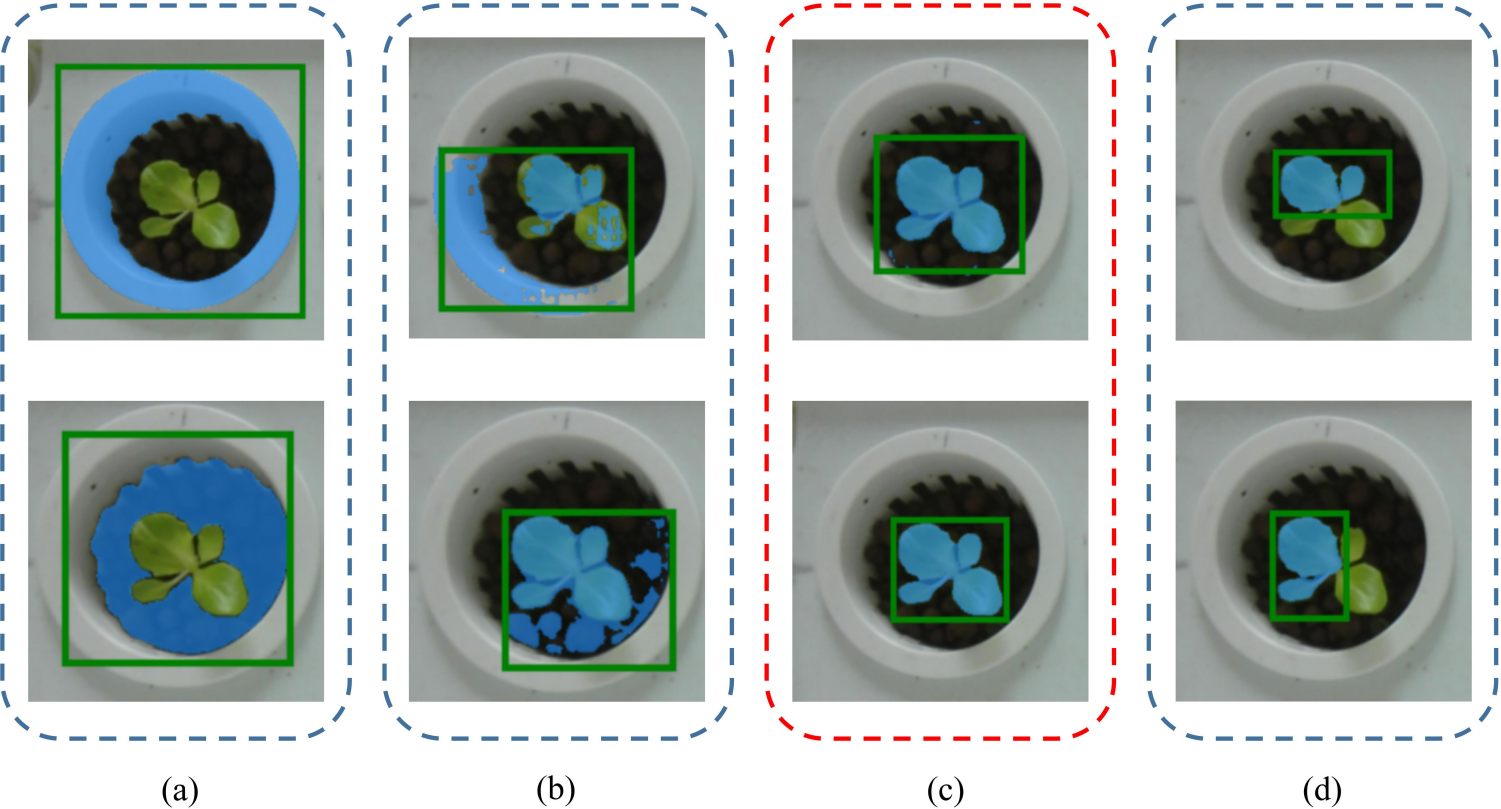
The impact of box prompts on SAM’s instance segmentation performance. Box prompts with excessive redundant space or those containing only part of the plant will reduce SAM’s ability to accurately segment the target plant instances.

Box prompts significantly influence SAM’s instance segmentation performance, including factors such as box position, size, and plant completeness. Among these, box position is crucial. Grounding DINO’s strong object detection capabilities enable accurate plant localization. However, the large number of redundant boxes it generates creates challenges in determining the optimal box size and ensuring the plant’s completeness. Excessive redundant space in the box can cause SAM to mistake background areas for the target region, resulting in oversized segmentation masks (**Figure 13b**) or misidentifying the background as the target (**Figure 13a**). Such errors can severely impact plant phenotyping. After removing redundant large boxes, ensuring the completeness of the target plant remains critical. If the box contains only part of the plant, SAM may fail to detect the remaining area (**Figure 13d**). To address these challenges, we propose the VC-NMS algorithm, which adopts a two-stage approach. In the first stage, NCGI values and confidence scores are combined (Equation 3), with higher penalties applied to boxes containing multiple nested boxes (Equation 4). This stage effectively leverages the green pixel information of hydroponic plants, preserving plant completeness while penalizing redundant large boxes. The second stage is similar to Soft-NMS but uses a Gaussian penalty function (Equation 6). Unlike Soft-NMS, we replace the traditional Intersection over Union (IoU) with Relative IoU (RIoU) (Equation 5), which effectively prevents small targets from being overlooked or incorrectly suppressed due to the presence of larger targets, thereby removing redundant boxes. VC-NMS significantly enhances the Average Precision of the final target bounding boxes, improving AP@0.5 by 0.177 over NMS and by 0.118 over Soft-NMS-L (**Table 3**), providing SAM with accurate and effective box prompts (**Figure 13c**).

### 4.2 Effect of point prompts on segmentation performance

The number and position of point prompts play a crucial role in the segmentation performance of SAM. To explore their impact and determine the optimal number and strategy of prompts, we conducted extensive comparative experiments. As shown in **Table 4**, SAM requires sufficient information to perform effective instance segmentation. A configuration of one positive and one negative point clearly does not provide enough information for SAM. However, SAM’s ability to process prompts is limited. When the number of positive and negative points exceeds two, the segmentation performance begins to degrade. This effect is particularly pronounced when multiple plant instances are present, further challenging SAM’s prompt processing capabilities. Based on the experimental results, we concluded that the optimal point prompt configuration consists of two positive points and two negative points. To determine the best locations for point prompts, we compared three different enhancement strategies: max entropy criterion, similarity maps with random sampling, and similarity maps with the max distance criterion. The max entropy method suffers from discontinuities in the green entropy map, resulting in significant gaps between regions. If negative points are placed in low-entropy areas of the plant, it severely hampers instance segmentation performance (**Figure 10a**). Similarity maps with random sampling can lead to point clustering, which wastes valuable prompt information and results in unstable points that are challenging to apply effectively (**Figure 10b**). In contrast, our approach—similarity maps with the max distance criterion—first selects high-quality base points using similarity maps and then applies the max distance criterion. This method effectively prevents point clustering while utilizing spatial information from the image more efficiently. As a result, the point prompts cover the entire plant area more effectively, yielding the best configuration (**Figure 10c**), which enables SAM to focus more accurately on the plant regions.

### 4.3 Generalization capability of zero-shot instance segmentation in vertical farming

In vertical farming, challenges such as varying light conditions, planting methods, plant types, camera perspectives, and image scales significantly complicate plant phenotyping and smart management. While traditional supervised instance segmentation models perform well for specific plants, they depend on costly, labor-intensive data collection and annotation, and struggle with new plants or complex environments. To tackle these challenges, we propose a Zero-shot Instance Segmentation framework using foundational models. Its key advantage is adaptability to various plant types and environmental conditions without requiring large annotated datasets. Our framework combines Grounding DINO and SAM, employing text, image, and spatial structure prompts (box and point prompts) to guide accurate plant instance segmentation. The open-set detection capability of Grounding DINO (text-prompted object localization) and SAM’s zero-shot segmentation generalization exhibit natural complementarity. This synergistic combination overcomes the predefined category limitation inherent in traditional instance segmentation approaches. As it does not require separate training for each plant, the framework quickly adapts to new plant varieties and growth stages, showcasing strong generalization capability. **Figure 11** illustrates that our zero-shot instance segmentation framework effectively handles segmentation tasks for various plants (e.g., lettuce and pakchoi) and growth stages (e.g., germination, seedling, rosette, and heading) in vertical farms. The framework maintains high segmentation accuracy across complex lighting, diverse backgrounds, and high-density planting scenarios, demonstrating adaptability to various plant species and environmental conditions. As shown in **Table 8**, while our model performs slightly lower than traditional supervised methods on the training set, it significantly outperforms them on both test sets. This approach addresses challenges in data collection and annotation, lowers the cost of plant phenotyping, and enhances efficiency for smart management in vertical farming.

## 5 Conclusions and future work

This study introduces a zero-shot plant instance segmentation framework that integrates Grounding DINO with SAM. SAM serves as the primary image segmentation model, leveraging box prompts generated by Grounding DINO with VC-NMS. Enhanced point prompts are created using the similarity maps combined with the max distance criterion. Finally, both box and point prompts are input into SAM’s prompt encoder to enable accurate instance segmentation, forming an automated zero-shot segmentation pipeline based on foundation models. The VC-NMS algorithm combines NCGI values with confidence scores, effectively eliminating redundant bounding boxes generated by Grounding DINO. The resulting plant instance bounding boxes exhibit significantly higher average precision compared to G-NMS and Soft-NMS methods. The similarity maps with max distance criterion ensure consistent prompt performance, allowing SAM to focus more effectively on the entire plant region. An optimal configuration of two positive and two negative points minimizes the adverse effects of excessive or insufficient prompts on SAM.

Experimental results indicate that our model surpasses SAM-based baseline models in segmentation performance, achieving an impressive IoU of 0.593 on the HP Dataset. Furthermore, the method exhibits remarkable zero-shot generalization ability, consistently achieving stable segmentation performance across diverse plant types, planting methods, viewing angles, and complex lighting conditions, highlighting its robustness. Compared to traditional supervised segmentation methods that depend on extensive annotated datasets, our approach achieves competitive performance while effectively extracting plant phenotypic features. The method effectively tackles the challenge of data scarcity in hydroponic image segmentation, offering robust support for intelligent detection in vertical farming.

The zero-shot capability enables our model to extend beyond vertical farming applications. Simple adaptation of Grounding DINO’s text_prompt allows direct deployment for phenotyping other green plants without training. Although our zero-shot instance segmentation framework has shown promising potential for application in vertical farming, it still faces certain limitations, such as real-time processing capabilities and segmentation accuracy in complex environments. To address these challenges, we plan to adopt a more lightweight SAM-based model (e.g., MobileSAMv2, Lite-SAM) to improve the framework’s real-time performance. In addition, by further optimizing the prompting strategy and incorporating more domain-specific agricultural knowledge (e.g., plant morphology priors for SAM decoder tuning), we aim to enhance the model’s segmentation accuracy in complex agricultural settings, thereby advancing the practical application of zero-shot instance segmentation in vertical farming.

## Data Availability

The original contributions presented in the study are included in the article/supplementary material. Further inquiries can be directed to the corresponding author.

## References

[B1] Abebe A. M. Kim Y. Kim J. Kim S. L. Baek J. (2023). Image-based high-throughput phenotyping in horticultural crops. Plants 12, 2061. doi: 10.3390/plants12102061 37653978 PMC10222289

[B2] Al-Kodmany K. (2018). The vertical farm: A review of developments and implications for the vertical city. Buildings 8, 24. doi: 10.3390/buildings8020024

[B3] Benke K. Tomkins B. (2017). Future food-production systems: vertical farming and controlled-environment agriculture. Sustainabil.: Sci. Pract. Policy 13, 13–26. doi: 10.1080/15487733.2017.1394054

[B4] Bodla N. Singh B. Chellappa R. Davis L. S. (2017). Soft-NMS--improving object detection with one line of code. In Proceedings of the IEEE international conference on computer vision (Venice, Italy), 5561–5569. doi: 10.1109/iccv.2017.593

[B5] Buxbaum N. Lieth J. H. Earles M. (2022). Non-destructive plant biomass monitoring with high spatio-temporal resolution via proximal RGB-D imagery and end-to-end deep learning. Front. Plant Sci. 13. doi: 10.3389/fpls.2022.758818 PMC9043900 35498682

[B6] Cardenas-Gallegos J. S. Severns P. M. Klimeš P. Lacerda L. N. Peduzzi A. Ferrarezi R. S. (2024). Reliable plant segmentation under variable greenhouse illumination conditions. Comput. Electron. Agric. 229, 109711–109711. doi: 10.1016/j.compag.2024.109711

[B7] Chang C.-L. Chung S.-C. Fu W.-L. Huang C.-C. (2021). Artificial intelligence approaches to predict growth, harvest day, and quality of lettuce (Lactuca sativa L.) in a IoT-enabled greenhouse system. Biosyst. Eng. 212, 77–105. doi: 10.1016/j.biosystemseng.2021.09.015

[B8] Chen T. Zhu L. Ding C. Cao R. Wang Y. Li Z.. (2023). SAM fails to segment anything? – SAM-adapter: adapting SAM in underperformed scenes: camouflage, shadow, medical image segmentation, and more. ArXiv (Cornell University). doi: 10.48550/arxiv.2304.09148

[B9] Dai H. Ma C. Liu Z. Li Y. Shu P. Wei X.. (2023). SAMAug: point prompt augmentation for segment anything model. ArXiv (Cornell University). doi: 10.48550/arxiv.2307.01187

[B10] Deng R. Cui C. Liu Q. Yao T. Remedios L. W. Bao S.. (2023a). Segment anything model (SAM) for digital pathology: assess zero-shot segmentation on whole slide imaging. ArXiv (Cornell University). doi: 10.48550/arxiv.2304.04155 PMC11971729 40190816

[B11] Deng G. Zou K. Ren K. Wang M. Yuan X. Ying S.. (2023b). SAM-U: multi-box prompts triggered uncertainty estimation for reliable SAM in medical image. Lect. Notes Comput. Sci., 368–377. doi: 10.1007/978-3-031-47425-5_33

[B12] Fan D.-P. Cheng M.-M. Liu Y. Li T. Borji A. (2017). “Structure-measure: A new way to evaluate foreground maps,” in Proceedings of the IEEE International Conference on Computer Vision (Venice, Italy), 4548–4557. doi: 10.1109/iccv.2017.487

[B13] Franchetti B. Ntouskos V. Giuliani P. Herman T. Barnes L. Pirri F. (2019). Vision based modeling of plants phenotyping in vertical farming under artificial lighting. Sensors 19, 4378. doi: 10.3390/s19204378 31658728 PMC6848939

[B14] Fu J. Yu Y. Li N. Zhang Y. Chen Q. Xiong J.. (2024). Lite-SAM is actually what you need for segment everything. Lect. Notes Comput. Sci., 456–471. doi: 10.1007/978-3-031-72754-2_26

[B15] Guo C. Pleiss G. Sun Y. Weinberger K. Q. (2017). On calibration of modern neural networks. ArXiv (Cornell University). doi: 10.48550/arxiv.1706.04599

[B16] Guo X. Qiu Y. Nettleton D. Schnable P. (2023). High-throughput phenotyping: A self-supervised sequential CNN method to segment overlapping plants. Plant Phenom. doi: 10.34133/plantphenomics.0052 PMC10194366 37213545

[B17] Guo X. Qiu Y. Nettleton D. Yeh C. Zheng Z. Hey S.. (2021). KAT4IA: K-means assisted training for image analysis of field-grown plant phenotypes. Plant Phenom. doi: 10.34133/2021/9805489 PMC8358166 34405144

[B18] Hwang Y. Lee S. Kim T. Baik K. Choi Y. (2022). Crop growth monitoring system in vertical farms based on region-of-interest prediction. Agriculture 12, 656. doi: 10.3390/agriculture12050656

[B19] Jiang Q. Huang Z. Xu G. Su Y. (2023). MIoP-NMS: Perfecting crops target detection and counting in dense occlusion from high-resolution UAV imagery. Smart Agric. Technol. 4, 100226. doi: 10.1016/j.atech.2023.100226

[B20] Kapur J. N. (1989). Maximum-Entropy models in science and engineering (New York: John Wiley & Sons).

[B21] Khan A. T. Jensen S. M. (2025). LEAF-net: A unified framework for leaf extraction and analysis in multi-crop phenotyping using YOLOv11. Agriculture 15, 196–196. doi: 10.3390/agriculture15020196

[B22] Kirillov A. Mintun E. Ravi N. Mao H. Rolland C. Gustafson L.. (2023). Segment anything. doi: 10.1109/ICCV51070.2023.00371

[B23] Lee U. Chang S. Putra G. A. Kim H. Kim D. H. (2018). An automated, high-throughput plant phenotyping system using machine learning-based plant segmentation and image analysis. PloS One 13, e0196615. doi: 10.1371/journal.pone.0196615 29702690 PMC5922545

[B24] Li C. Adhikari R. Yao Y. Miller A. G. Kalbaugh K. Li D.. (2020). Measuring plant growth characteristics using smartphone based image analysis technique in controlled environment agriculture. Comput. Electron. Agric. 168, 105123. doi: 10.1016/j.compag.2019.105123

[B25] Li Y. Wang D. Yuan C. Li H. Hu J. (2023). Enhancing agricultural image segmentation with an agricultural segment anything model adapter. Sensors 23. doi: 10.3390/s23187884 PMC10534855 37765940

[B26] Lin Z. Fu R. Ren G. Zhong R. Ying Y. Lin T. (2022). Automatic monitoring of lettuce fresh weight by multi-modal fusion based deep learning. Front. Plant Sci. 13. doi: 10.3389/fpls.2022.980581 PMC9458202 36092436

[B27] Liu H. Xu Z. (2023). Editorial: Machine vision and machine learning for plant phenotyping and precision agriculture. Front. Plant Sci. 14. doi: 10.3389/fpls.2023.1331918 PMC10711600 38089787

[B28] Liu S. Zeng Z. Ren T. Li F. Zhang H. Yang J.. (2023). Grounding DINO: marrying DINO with grounded pre-training for open-set object detection. doi: 10.48550/arxiv.2303.05499

[B29] Ma J. Wang B. (2023). Segment anything in medical images. ArXiv. doi: 10.48550/arXiv.2304.12306 PMC10803759 38253604

[B30] Margolin R. Zelnik-Manor L. Tal A. (2014). How to evaluate foreground maps. IEEE Xplore. doi: 10.1109/CVPR.2014.39

[B31] Neubeck A. Van Gool L. (2006). Efficient non-maximum suppression. IEEE Xplore. doi: 10.1109/ICPR.2006.479

[B32] Otsu N. (1979). A threshold selection method from gray-level histograms. IEEE Trans. Sys. Man Cybernet. 9, 62–66. doi: 10.1109/tsmc.1979.4310076

[B33] Ren T. Liu S. Zeng A. Lin J. Li K. Cao H.. (2024). Grounded SAM: assembling open-world models for diverse visual tasks. ArXiv.org. doi: 10.48550/arXiv.2401.14159

[B34] Reyes-Yanes A. Martinez P. Ahmad R. (2020). Real-time growth rate and fresh weight estimation for little gem romaine lettuce in aquaponic grow beds. Comput. Electron. Agric. 179, 105827. doi: 10.1016/j.compag.2020.105827

[B35] Trivedi M. Gupta A. (2021). Automatic monitoring of the growth of plants using deep learning-based leaf segmentation. Int. J. Appl. Sci. Eng. 18, 1–9. doi: 10.6703/IJASE.202106_18(2).003

[B36] Wang X. Zhang R. Kong T. Li L. Shen C. (2020). SOLOv2: dynamic and fast instance segmentation. ArXiv.org. doi: 10.48550/arXiv.2003.101520/arXiv.2003.101522

[B37] Wong C. E. Teo Z. W. N. Shen L. Yu H. (2020). Seeing the lights for leafy greens in indoor vertical farming. Trends Food Sci. Technol. 106, 48–63. doi: 10.1016/j.tifs.2020.09.031

[B38] Wu J. Zhang Y. Fu R. Fang H. Liu Y. Wang Z.. (2023). Medical SAM adapter: adapting segment anything model for medical image segmentation. ArXiv.org. doi: 10.48550/arXiv.2304.12620 40121809

[B39] Zhang C. Han D. Qiao Y. Kim J. U. Bae S.-H. Lee S.. (2023a). Faster segment anything: towards lightweight SAM for mobile applications. ArXiv (Cornell University). doi: 10.48550/arxiv.2306.14289

[B40] Zhang C. Han D. Zheng S. Choi J. Kim T.-H. Hong C. S. (2023b). MobileSAMv2: faster segment anything to everything. ArXiv (Cornell University). doi: 10.48550/arxiv.2312.09579

[B41] Zhang J. Ma K. Kapse S. Saltz J. Vakalopoulou M. Prasanna P.. (2023d). SAM-Path: A segment anything model for semantic segmentation in digital pathology. Lect. Notes Comput. Sci., 161–170. doi: 10.1007/978-3-031-47401-9_16

[B42] Zhang C. Puspitasari F. D. Zheng S. Li C. Qiao Y. Kang T.. (2023c). A survey on segment anything model (SAM): vision foundation model meets prompt engineering. ArXiv.org. doi: 10.48550/arXiv.2306.06211

[B43] Zhang L. Xu Z. Xu D. Ma J. Chen Y. Fu Z. (2020). Growth monitoring of greenhouse lettuce based on a convolutional neural network. Horticult. Res. 7. doi: 10.1038/s41438-020-00345-6 PMC7395764 32821407

[B44] Zhou J. Applegate C. Alonso A. D. Reynolds D. Orford S. Mackiewicz M.. (2017). Leaf-GP: an open and automated software application for measuring growth phenotypes for arabidopsis and wheat. Plant Methods 13. doi: 10.1186/s13007-017-0266-3 PMC5740932 29299051

